# Ferroptosis inhibition protects against α-synuclein-related neuronal cell death

**DOI:** 10.1038/s41419-025-08319-z

**Published:** 2025-12-14

**Authors:** Naďa Majerníková, Maria J. Caiado, Renée I. Seinstra, Suzanne Couzijn, María E. Goya, Casandra Salinas Salinas, Hannah Truong, Tineke van der Sluis, Anneke Miedema, Leon C. L. T. van Kempen, Gawain McColl, Ellen A. A. Nollen, Amalia M. Dolga, Wilfred F. A. den Dunnen

**Affiliations:** 1 Department of Molecular Pharmacology, Groningen Research Institute of Pharmacy, University of Groningen, Groningen, the Netherlands; 2 Department of Pathology and Medical Biology, University Medical Centre Groningen, University of Groningen, Groningen, the Netherlands; 3 Research School of Behavioral and Cognitive Neuroscience, University of Groningen, Groningen, the Netherlands; 4 Research Institute Mechanisms of Health, Ageing and Disease (MoHAD), University Medical Centre Groningen, Groningen, the Netherlands; 5 European Research Institute for the Biology of Ageing, University of Groningen, University Medical Centre Groningen, Groningen, the Netherlands; 6 The Florey Institute of Neuroscience and Mental Health, The University of Melbourne, Parkville, VIC Australia; 7 Department of Pathology, Maastricht University Medical Centre, Maastricht, the Netherlands

**Keywords:** Cell death in the nervous system, Parkinson's disease

## Abstract

Parkinson’s disease (PD), characterized by α-synuclein (α-syn) pathology, affects millions of people worldwide. While current treatments mainly symptomatically address the motor aspects of PD, they lack efficacy in delaying or halting the degenerative process. Ferroptosis, a type of programmed cell death characterized by iron-dependent lipid peroxidation, has been previously linked to PD. Advancing the development of neuroprotective treatments hinges on comprehending the interplay between PD’s pathological hallmarks and cell death. We examined six ferroptosis-related markers (ferroportin, ferritin, NCOA4, cytochrome c, GPX4, and 4HNE) in mesencephalic tissues from 10 PD patients and 11 age-matched controls. In post-mortem brains of controls, several ferroptosis-related markers were differentially expressed in functional subregions of the substantia nigra (SN), suggesting differential ferroptosis vulnerability. Moreover, ferritin and ferroportin levels were reduced in relation to α-synuclein pathology, indicating impaired iron storage and export, and suggesting increased vulnerability to ferroptosis in Parkinson’s disease. Additionally, using digital spatial transcriptomics, we revealed ferroptosis-related differentially expressed genes (DEGs) in PD, which altogether pointed towards higher ferroptosis vulnerability in PD compared to control brains. To support our post-mortem findings, we used in vitro models (LUHMES neurons and mouse cortical neurons (PCNs)) and an α-syn overexpression *C. elegans* model. Co-treatment with low concentrations of α-syn and RSL3, which alone did not cause cell death, increased neuronal vulnerability to cell death, which was mitigated by ferrostatin-1 (Fer-1) but not deferoxamine (DFO) in cortical and dopaminergic neurons. Finally, α-syn expression in *C. elegans* increased iron levels, exacerbated by ferritin knockdown and reduced by DFO, which decreased α-syn inclusions. These results indicate that α-syn-related cell death can be altered by ferroptosis inhibition, and targeting the ferroptosis pathway could reduce or slow cell death associated with PD pathology. However, ferroptosis vulnerability appears cell- and model-dependent, suggesting effective therapeutic strategies may require a more comprehensive approach, targeting multiple aspects of the pathway while considering timing to achieve optimal outcomes.

## Introduction

Parkinson’s disease (PD) has emerged as a growing global health concern, gaining significant attention and research into elucidating the molecular pathways leading to neurodegeneration and PD-related symptoms [1]. This progressive neurodegenerative disorder is characterized by distinct pathological features, including the accumulation of iron in the substantia nigra pars compacta (SNc) and the aggregation of α-syn within Lewy bodies, ultimately leading to the degeneration of dopaminergic neurons [2, 3]. PD gives rise to a spectrum of motor and non-motor symptoms, encompassing bradykinesia, rest tremors, mental health challenges, and sleep disturbances [4]. Unfortunately, current therapeutic approaches primarily address motor symptoms, leaving the underlying neurodegenerative process unaddressed. To bridge this therapeutic gap, it is imperative to gain a deeper understanding of the complex interplay between PD’s pathological hallmarks and cell death mechanisms.

Cell death can be categorized into accidental (ACD) or regulated cell death (RCD) [5]. PD-related neuronal death was initially linked to apoptosis, a common RCD [6]. With new insight into PD mechanisms like mitochondrial dysfunction, immune activation, lipid dysregulation, and metal ion imbalance, cell death was also attributed to necrosis [7]. Recent findings show that multiple cell death pathways are likely to contribute to neuronal loss [8, 9]. Ferroptosis, a form of RCD driven by iron-dependent lipid peroxidation, is distinct from apoptosis, necrosis, and autophagy [10, 11]. First characterized by Dixon and colleagues in 2012 [10], ferroptosis emerged as a pivotal player in the realm of PD pathology [12]. While our bodies naturally absorb iron through dietary intake, the absence of efficient excretion routes necessitates rigorous cellular mechanisms to maintain iron concentrations and prevent the onset of ferroptosis. Key components in this delicate balance include transferrin-1, facilitating iron entry into cells to meet cellular demands, and ferroportin, enabling iron export and safeguarding against ferroptotic cell death [11, 13].

Within the context of PD, the pathways of ferroptosis intersect with the disease’s pathogenesis [8, 11], making it essential to explore the factors contributing to iron homeostasis dysregulation and the subsequent initiation of ferroptosis. Aging and age-related diseases introduce vulnerabilities into these finely tuned mechanisms [14, 15], potentially resulting in heightened intracellular iron levels. This disruption sets off a cascade of events, including mitochondrial damage, ferritinophagy, and ferroportin dysfunction, culminating in elevated intracellular iron concentrations, reactive oxygen species (ROS) accumulation, mitochondrial dysfunction, and lipid peroxidation, which are also distinctive features of ferroptosis [11, 16, 17].

The characteristic pathological changes seen in PD, such as elevated lipid peroxidation products and defects in the system xc−/GSH/GPX4 antioxidative axis, align well with the ferroptosis cell-death pathway [18, 19]. The finding of iron and α-syn coexistence in Lewy bodies in the SNc in postmortem analyses from PD patients solidified the link between neurodegenerative disease and ferroptosis [20]. Ferroptosis involvement in dopaminergic cell death has been demonstrated in in vitro and in vivo models of PD. Lund human mesencephalic (LUHMES) cells treated with erastin were protected by the iron chelator deferiprone (DFP) and the antioxidant N-acetylcysteine (NAC). Additionally, in the 1-methyl-4-phenyl-1,2,3,6-tetrahydropyridine (MPTP) mouse model, a well-known PD animal model, the MPTP toxicity was reduced by Fer-1, the lipid peroxidation inhibitor [21]. More recent findings in pluripotent stem cell (iPSC)-derived neurons with triplication of the α-syn-encoding gene SNCA also showed that the interaction between α-syn fibrils and membranes induced lipid peroxidation, which was prevented by ferroptosis inhibitors [22]. Iron chelators and other ferroptosis inhibitors have shown improvements in PD pathologies and provide neuroprotection in in vitro and in vivo models of PD; nevertheless, the translation of these models has not reached a viable therapy. First, the FAIR-PARK randomized, double-blinded, placebo-controlled study pointed to the potential efficacy of deferiprone (DFP) in reducing brain iron levels, which were thought to contribute to the progression of PD [23] (NCT00943748). Later, a phase 2 clinical trial of DFP in PD patients revealed symptoms worsening measured by the Movement Disorder Society-sponsored revision of the unified Parkinson’s disease rating scale (MDS-UPDRS) (the mean total score at baseline was 34.3 and increased 15.6 points, compared to a 6.3-point increase of the placebo group) [24]. Even though improvements in the discoveries of this vicious cycle between iron and α-syn have been reported, the causal relationship and molecular mechanisms need to be further studied to bridge the clinical gap of anti-ferroptotic treatments.

To better understand the relationship between α-syn and ferroptosis, we aimed to investigate whether the ferroptosis-related markers co-localize with α-syn inclusions of PD dopaminergic neurons in comparison with dopaminergic neurons without α-syn inclusions of age-matched controls and of PD patients. To further investigate the interplay between α-syn and ferroptosis vulnerability, we used various in vitro models, such as primary mouse cortical neurons (PCNs) and human differentiated dopaminergic LUHMES cells [25, 26]. We next challenged them with α-syn fibrils and RSL3 in the presence or absence of ferroptosis inhibitors deferoxamine (DFO) and ferrostatin-1 (Fer-1). A key aim was to compare DFO and Fer-1 treatments, as they target different parts of the ferroptotic pathway. Furthermore, we used an α-syn overexpression model in *C. elegans* to investigate the potential of ferroptosis inhibitors on α-syn-related toxicity. Our findings indicate that α-syn alters the expression of ferroptosis-related markers in the human brain and that co-treatment of α-syn fibrils and RSL3 increases cell death of both neuronal cell types. Ferroptosis attenuation decreased α-syn-induced cell death in vitro and protected against α-syn-related toxicity in vivo, suggesting that the ferroptosis pathway contributes to PD pathology, and improving their targeting approaches might alleviate PD pathology and symptoms.

## Material and methods

### Subjects

For this study, 10 patients with PD and 11 age-matched controls without neurodegenerative disease were selected (Supplementary Fig. 1). The formalin-fixed, paraffin-embedded (FFPE) tissues used for this study were obtained during routine neuropathological diagnostics. The 21 subjects in this study are part of the larger BRAIN-cohort (Brain Research in Aging Inflammation and Neurodegeneration). The use of post-mortem brain tissues from the BRAIN-cohort was evaluated by the Medical Ethical Evaluation Committee (METc nr 2021/496) on 31 August 2021. Based on the submitted documents, the METc UMCG concluded that the described protocol is not clinical research with human subjects as defined by the Medical Research Involving Human Subjects Act (WMO). The LTc UMCG (local review board for non-WMO research) positively reviewed the described protocol (research registration number 202100608). Information regarding age, post-mortem delay, and fixation time was collected per patient, which was needed to perform the correlation and multivariate analysis in Prism 8.4.2 (GraphPad Software, USA) (Supplementary Fig. 1, Supplementary Table 1), as well as a multiple linear regression analysis (MLR) using IBM SPSS (28.0.1.0) (Supplementary Fig. 2).

### Immunohistochemistry

The immunohistochemistry protocol was adapted from Seidel and colleagues (2012). FFPE blocks were cut into histological sections (3μm thick) sequentially across the mesencephalon (dopaminergic neurons in the SNc) and then fixed on adhesive Starfrost glass slides. The slides were first deparaffinized using a standard protocol of 2× 10 min xylol washes, followed by rehydration in graded alcohol solutions of 100%, 96% and 70% ethanol. After washing with demineralized water, the sections underwent an antigen retrieval step in microwave-warmed (300 W) 0.1 M Tris/hydrochloride (HCl) buffer (pH 9.0), for 15 min, followed by a cooling-down period at room temperature (RT). The slides were then soaked for 30 min in a solution of diluted (0.3%) hydrogen peroxide (H_2_O_2_) in phosphate-buffered saline (PBS) to block endogenous peroxidase. After washing, sections were blocked with the Avidin/Biotin Blocking Kit (Vector Laboratories, SP-2001) if GARbio/RAGbio secondary antibodies were used (ferroportin and GPX4) (Vector Laboratories SP-2001/ZG1009). The slides were incubated with rabbit or goat primary polyclonal antibody, diluted in 1% bovine serum albumin (BSA)/PBS (60 min, RT unless stated otherwise) (Supplementary Table 2). After three times PBS washing steps, the slides were incubated for 30 min at RT with horseradish peroxidase-labeled secondary antibody (goat-anti-rabbit (GAR^PO^) or rabbit-anti-goat (RAG^PO^)) (Supplementary Table 2) diluted in 1% BSA/PBS + 1% normal human (AB) serum to block non-specific binding. The slides were then washed again 3× with PBS and incubated for another 30 min in the corresponding tertiary antibody (Supplementary Table 2), also diluted in 1% BSA/PBS + 1% normal human (AB) serum. After 3× PBS washes, the slides were stained for 10 min with 3,3-diaminobenzidine (DAB) (Sigma) in a diluted solution of (0.03%) H_2_O_2_ in PBS for 10 min. The slides were then washed three times with demineralized water, counterstained with haematoxylin and eosin (HE) for 2 min, and then washed with tap water. Finally, the slides were dehydrated in sequentially increasing concentrations of ethanol (70%, 96% and 100%). The sections were dried overnight and mounted in medium and coverslips in the Sakura Tissue-Tek® Prisma^TM^. For α-syn staining, sections were deparaffinized with decreasing xylol and ethanol. Antigen retrieval involved submersion in 0.1 M Tris/HCl buffer (pH 9.5), heated in the microwave, followed by transfer to 98% formic acid for 3 min. Endogenous peroxidase was blocked with diluted (0.03%) H_2_O_2_ in PBS for 30 min at RT. Using the BenchMark (Ventana) for automated immunohistochemistry, sections were incubated for 1 h with the primary antibody in 1% BSA/PBS (Supplementary Table 2). After 3× PBS washes, slides were incubated for 30 min with the corresponding secondary antibody (Supplementary Table 2) at RT, washed, and immunoprecipitated with DAB as the chromogen. All sections were counterstained in HE, rehydrated, and mounted in medium with a cover slip. The sections were then imaged using the Hamamatsu NanoZoomer 2.0HT. Finally, a total of 73 slides (Supplementary Table 1) were stained for HE; this was done automatically in the Sakura Tissue-Tek® PrismaTM. This stain serves as a control for the neuromelanin dark pigmentation, which is independent of the dark-pigmented DAB stain.

### Positive pixel density scoring

Pixel-density scoring analysis was carried out using Aperio ImageScope (Leica Biosystems) v.12.4.3.5008, to quantify pathology and ferroptosis-related markers’ expression. To make a fair selection for the analysis, an inclusion criterion was developed, whereby, per patient scan, a total of 10 neurons with α-syn inclusions and 10 neurons without α-syn inclusions were selected throughout the SNc (Supplementary Fig. 3). Within the patient groups with PD, for the pathology-negative neurons, the selection was based on the presence of a nucleus, neuromelanin stain (as a control for the right location, i.e. the SNc), and the rough endoplasmic reticulum marked by Nissl staining, alongside no signs of α-syn inclusions. These components were present in at least one of the slides for a given cell. All pathology-positive neurons were selected using the same criteria as those for detecting dopaminergic cells, except for those in which α-syn inclusion has been studied. In age-matched controls cases, we divided the SNc into three anatomical areas according to physiological function: lateral, ventral and medial SNc (Fig. 2A). This selection was not possible for PD patients due to their end-stage disease profile and extensive neurodegeneration, therefore cells had to be selected primarily from the ventral area and supplemented with cells from either medial or lateral SNc areas to reach a minimum count of 10 (Supplementary Fig. 3). As the control group does not show pathological hallmarks, only 10 cells per anatomical SNc area were selected per patient. In the present investigation, only alive cells with inclusion were considered, thereby excluding free protein aggregates resulting from cell death.

**Fig. 1:**
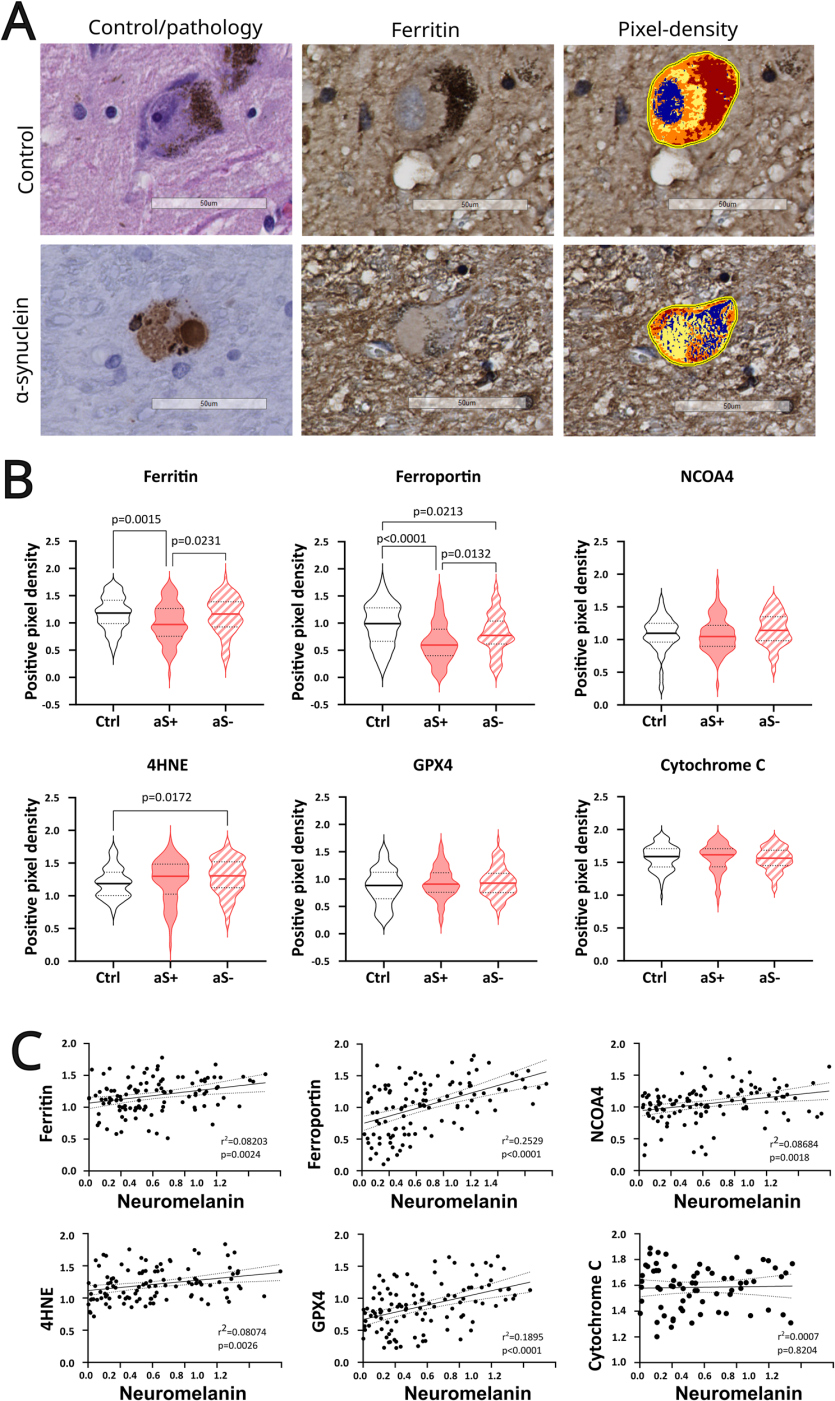
The expression of ferroptosis-related proteins is changed in post-mortem PD brain. **A** Histomicrographs of dopaminergic SN cells with (d) and without (a) α-syn pathology (left column), with their respective ferritin stain (middle column), followed by the pixel-density contrast image produced on Leica ImageScope (right column). For our analysis, we used “Np = number of positive” (orange), “Nsp = number of strong positive” (red), and “Ntotal = total number (positive + negative)” of pixels from the results table. To obtain our normalized raw data, we then input these values into the following equation: ((*N*_p_) + (2 × *N*_sp_))/ *N*_total_. **B** Violin plots illustrate the expression of ferroptosis-related markers in α-syn positive cells, which were compared to cells from age-matched controls and α-syn negative cells from PD patients. Statistical significance was determined using an ANOVA with Sidak correction for multiple comparisons for ferritin, while the remaining markers were analyzed using a Kruskal–Wallis test with Dunn’s post hoc test. The pixel densities of ferritin and ferroportin were significantly lower in α-syn positive cells (and α-syn negative cells only for ferroportin) compared to brain cells of age-matched controls (ferritin: *p* = 0.0015, ferroportin: *p* < 0.0001, *p* = 0.0213). Ferritin and ferroportin pixel densities were significantly higher in α-syn negative cells compared to α-syn positive cells in the brains of PD patients (*p* = 0.0231, *p* = 0.0132, respectively). 4HNE pixel density was higher in α-syn negative cells compared to brain cells of age-matched controls (*p* = 0.0172). **C** A scatter plot with simple linear regression illustrates the level of correlation between neuromelanin and each ferroptosis-related marker expression. All ferroptosis-related markers’ expressions show a positive correlation (Spearman’s correlation test) with neuromelanin. However, these correlations are weak (simple linear regression, *r*^2^ < 0.6000), and none of them are statistically significant (simple linear regression, *p* < 0.05).

After selecting the pathology-positive and -negative neurons in each scan, the same cells were selected in the HE slides and matched to the slides stained for ferroptosis-related markers (Fig. 1A). In the age-matched control group, the cells were selected directly from the HE stain and then matched to the adjacent marker stain (Supplementary Table 1). Afterwards, pixel-density scoring was used to quantify the expression of the six ferroptosis markers: ferritin, ferroportin, nuclear receptor co-activator 4 (NCOA4), 4-hydroxy-2-nonenal (4HNE), glutathione peroxidase 4 (GPX4), and cytochrome c. In the initial analysis, the expression of these markers was quantified in neurons from the ventral SNc of age-matched controls and compared to neurons with and without pathological inclusions from the SNc of PD patients (primarily ventral, but also containing medial and lateral SNc). In the second analysis, neurons from age-matched controls were compared between ventral, medial, and lateral anatomical areas of the SNc to explore variability in ferroptosis vulnerability within the SNc. Each cell was analyzed individually by manual selection, using the “View>Annotations” and pen tools on Aperio Leica ImageScope. For measurement values, we selected “View>Analysis>Choose Algorithm…>Positive Pixel Count v9”. In the output channel, all values were kept as default, in the input dropdown menu, “Iwp(High)”, “Iwp(Low)” and “Ip(Low)” were set as specific threshold settings for each marker (Supplementary Table 3). All other settings were left as default. Every time the settings were changed for different markers, we first selected “Tune Algorithm” to check that the thresholds were correct. Then, we selected “Analyze” to obtain the results table. Pixel-density scores were calculated using the following equation:

$$\begin{array}{rcl}\begin{array}{c}\begin{array}{c}(mediumintensitypixelation)+(highintensitypixelationx2)\\ totalselectedareapixilation\,\end{array}\end{array}\end{array}$$

where by “*N*_p_ = number of positives” (orange) corresponds to medium-intensity pixels, “*N*_s__p_ = number of strong positives” (red) corresponds to high-intensity pixels, and “*N*_total_ = total number (positive+negative)” corresponds to total pixels in the selected area (cell of interest) (Fig. 1A). We multiply high-intensity pixel values by two to account for the stronger positive pixels since the results from the program are representative of the area covered by the pixels with higher intensity, but do not account for the actual higher intensity value. At the end, a range of 75–110 cells was used in each analysis. This variable range results from practical acquisition errors that affect the visualization and selection of the cells of interest. The average cell size of all cells ranged from 10,804 to 14,819 pixels, indicating that cell size was not likely to affect the measurements taken. Nonetheless, all pixel-density calculations were normalized to total cell size since the 3D cells were measured in the 2D plane from different layered cuts.

### Probe hybridization and TH staining for nanostring GeoMx digital spatial profiler (DSP)

**Fig. 2:**
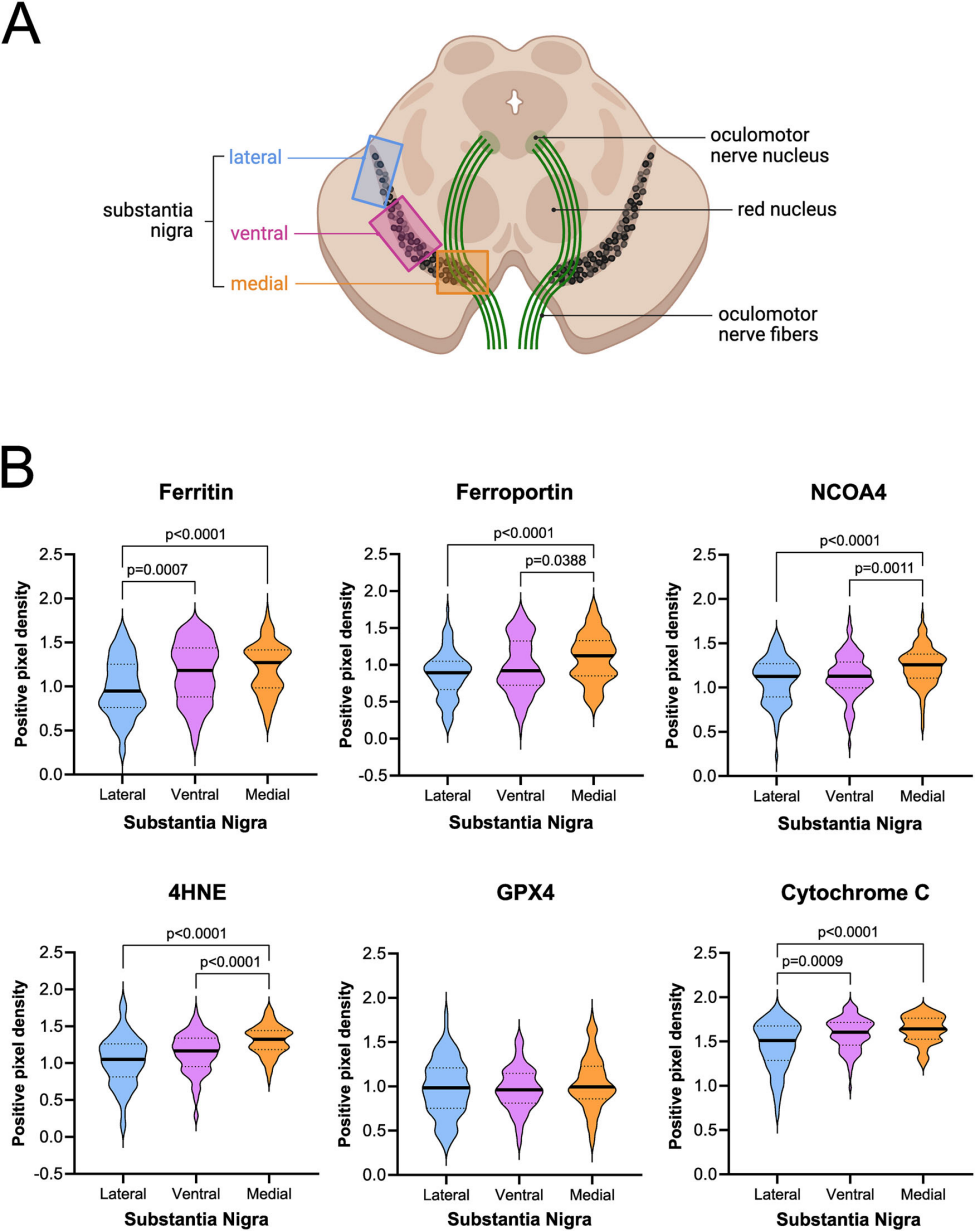
The expression of ferroptosis-related proteins is different between SN subregions in the post-mortem brain of controls. **A** Schematic to show how the SN pars compacta (SNc) was divided into three subregions: lateral (blue), ventral (pink), and medial (orange) using fiber tracts and physiological functions as guidelines. **B** Violin plots illustrate the expression of ferroptosis-related markers in the three different subregions of the SNc within the mesencephalon of post-mortem control cases. Statistical significance was determined using an ANOVA with Sidak correction for multiple comparisons for GPX4, while the remaining markers were analyzed using a Kruskal–Wallis test with Dunn’s post hoc test. The pixel densities of ferritin and cytochrome c were significantly higher in medial SNc compared to the lateral SNc (*p* < 0.0001), and the expression in ventral was also higher compared to the lateral SNc (ferritin: *p* = 0.0007, cytochrome c: *p* = 0.0009). Ferroportin, NCOA4, and 4HNE showed higher positive pixel densities in medial SNc compared to ventral (ferroportin: *p* = 0.0388, NCOA4: *p* = 0.0011, 4HNE: *p* < 0.0001) and compared to the lateral subregion (*p* < 0.0001).

A tissue microarray (TMA) block was constructed with FFPE tissues from the substantia nigra (sectioned at 5 μm) from PD patients (*n* = 8, with ~4 cores/patient) and age-matched controls (*n* = 11, with ~4 cores/individual), using a microtome and mounted on Superfrost Plus slides (Cat#12-550-15, Fisher Scientific) as per Nanostring’s instructions (Fig. 2A). The TMA slide was baked at 60 °C for 2 h before processing. Then, the slide was deparaffinised using xylene and rehydrated in a series of 100% ethanol, and 95% ethanol, followed by antigen retrieval with 1X Tris/ethylenediaminetetraacetic acid (EDTA) (pH 9) (Cat#11280, SERVA) at 99 °C for 18 min using a steamer. To expose the RNA targets, the TMA was then incubated in PBS/Proteinase K solution (Cat#100005393, Invitrogen) (1 µg/ml) for 15 min, followed by fixation in neutral buffered formalin (pH 7). For hybridization, Nanostring Whole Transcriptome Atlas probes (Cat#121401102, Nanostring) were applied, and the slide was incubated overnight at 37 °C in a humid environment using a HybridEZ oven. Excess probes were washed off with 2x saline sodium citrate (SSC) (Cat#15557-044, Invitrogen) and deionised formamide (Cat#AM9342, Ambion), followed by a 30 min incubation with “buffer W” (#121300313, Nanostring slide preparation kit) to block nonspecific signal. To target dopaminergic neurons, the TMA was then stained with 1:200 primary anti-Tyrosine Hydroxylase (TH) antibody (Cat#GTX113016, Genetex), combined with 1:10 SYTO13 nuclear stain (Cat# 121300303, Nanostring) (directly conjugated to a FITC fluorescent label) for 60 min at room temperature (RT). Excess antibodies were washed off with 2×SSC, and the slide was then incubated in 1:100 secondary antibody donkey-anti-rabbit, conjugated to Alexa Fluor® F647 fluorochrome (Cat#A31573, Invitrogen) for 30 min at RT.

### Imaging, probe collection, and library preparation

GeoMx-NSG data was prepared and primarily analyzed in R-studio (v4.3.2) using Nanostring-provided packages GeoMxTools (version 3.7.3) and NanoStringNCTools (v1.10.1). Quality control (QC) was performed to ensure sequencing quality and sampling adequacy. Particularly, ROI were segmented based on a minimum TH-positive area of 1000 µm^2^. Segments failing the QC criteria were removed from the dataset, and probe-level QC was performed to remove low-performing probes, yielding a total of 17,274 detected genes. Then, Quartile 3 (Q3) normalization was applied to account for variations in data distribution across segments and ensure appropriate comparisons between samples. To identify differentially expressed genes (DEGs), the Limma package (v3.58.1) was employed, for its suitability in microarray studies. A linear model (with empirical Bayes smoothing) was fit to the expression data using a design matrix for group comparisons, which allowed for the estimation of gene expression levels in PD vs Control. For this analysis, a ferroptosis-related gene list containing 76 genes was created based on the current literature and human gene set databases, including Gene Set Enrichment Analysis (GSEA) and Kyoto Encyclopedia of Genes and Genomes (KEGG) (Supplementary Table 4). Ferroptosis-related DEGs were then visualized in a volcano plot with log-fold change (logFC) of 0.6 and adjusted p < 0.05.

### Chemical compounds

RSL3 was purchased from Selleckchem, Netherlands (Cat. No. S8155); Fer-1 from Sigma-Aldrich, USA (Cat. no. 347174-05-4); DFO from Tocris Bioscience, USA (Cat. no. 5764). Α-syn 61-140 was purchased from Rpeptide, USA (Cat. ID S-1013-1) as well as from Origene, USA (Cat. No. SA6006X). Stock solutions were prepared and dissolved following the manufacturer's instructions. Corresponding volumes of the stock solutions were diluted in culture medium to reach the final concentrations listed, and culture medium was used as a vehicle. A pre-treatment of inhibitors was performed for 1 h and/or 4 h (as indicated in the figure legends), followed by treatment and/or co-treatment for 24 h with α-syn in all experiments, unless otherwise indicated.

### Cell culture

PCNs were isolated from embryonic (E13-14) C57BL/6 mice cultured at 37 °C in a 5% CO_2_ atmosphere and 95% humidity on PEI (polyethylenimine) coated (Cat. No. P3143, Sigma-Aldrich, USA) 8 well Ibidi plates (60,000 cells per well) in neurobasal medium (Cat. No. 2508186, Gibco, Thermo Fisher Scientific, USA) with 2 mM L-glut and 2% B-27 supplement [27] (Cat. No. 17504044; Gibco, Thermo Fisher Scientific, USA). These experiments are approved and conducted according to the rules of the Animal Experiments Committee (DEC) at the University of Groningen. All media was supplemented with 1% penicillin and streptomycin (Cat. No. 5070-063; Gibco, Thermo Fisher Scientific, USA) and was replenished every 2–3 days. Treatments were done at day 7 unless otherwise indicated.

LUHMES (Lund human mesencephalic) cells [25, 26] (originally provided by Marcel Leist lab) were cultured at 37 °C in a 5% CO_2_ atmosphere and 95% humidity in T25 flasks (Cat. No. 169900, Thermo Fisher Scientific, USA). For fluorescent imaging experiments, cells were seeded in eight-well Ibidi plates (Cat. No. 80826, Ibidi GmbH, Germany) that were coated overnight with Poly-L-lysine (0.44 mg/ml (PLL), Cat. No. P4707, Sigma-Aldrich, USA) and fibronectin solution (0.001 mg/ml, Cat. No. F1141, Sigma-Aldrich, USA). Flasks and Ibidi plates were washed with phosphate-buffered saline (PBS) before cell seeding. Proliferating cells were maintained in T25 flasks in advanced Dulbecco’s modified Eagle’s medium (DMEM)/F12 (Cat. No. 12634010 Gibco, Thermo Fisher Scientific, USA) supplemented with 1% N-2 (Cat. No. 17502048 Gibco/Invitrogen, Thermo Fisher Scientific, USA), 2 mM L-Glutamin (L-glut) (Cat. No. G7513, Sigma-Aldrich, USA) and 0.04 µg/ml basic fibroblast growth factor (bFGF) (Cat. No. 4114-TC, R&D Systems, USA). For the dopaminergic neuron differentiation, following 24 h of cell seeding, the proliferative media was replaced by differentiation media composed of advanced DMEM/F12 with 1% N2-supplement, 2 mM L-glut, 1 µg/ml tetracycline (Cat. No. T-7660, Sigma-Aldrich, USA), 1 mM dibutyryl cyclic AMP (Cat. No. D0627, Sigma-Aldrich, USA) and 2 ng/ml recombinant human glial cell-derived neurotrophic factor (GDNF) (Cat. No. 212-GD, R&D Systems, USA). Following two days of pre-differentiation and at ~80% confluency, cells were harvested with Trypsin (Cat. No. 25300062, Gibco, Thermo Fisher Scientific, USA), centrifuged for 3 min at 300×*g,* and seeded at 100,000 cells per well in 8-well Ibidi plates. LUHMES cells were left to fully differentiate for an additional 4 days, and we started with various treatments at day 7 of differentiation.

### Cell viability

Following 24 h of various treatments, differentiated LUHMES cells and PCNs in 8 well Ibidi plates were incubated with 1.5 µg/ml Hoechst 33342 (Cat. No. 33342, Invitrogen, Thermo Fisher Scientific, USA) for 30 min to stain nuclei, and then with 1 µg/ml propidium iodide (PI) (Cat. No. V13242, Invitrogen, Thermo Fisher Scientific, USA) to identify cells with damaged membranes as PI is not permeant to live cells. PI staining was performed right before imaging to avoid false positives that could occur at longer incubation times. Images were captured using live cell-imaging fluorescence Nikon Inverted Research Microscope ECLIPSE Ti2-E/Ti2-E/B at ×40 magnification, Hoechst 33342 (excitation 350 nm, emission 461 nm), and membrane-compromised cells stained with PI (excitation 570 nm, emission 602 nm) from 8 to 10 fields per well. Cell count was performed using the cell counter plug-in of ImageJ. PI positivity is depicted as a percentage of PI-positive cells in the total number of cells. N represents the number of independent experiments, and graphed values are from representative individual technical replicates.

### *C. elegans* strains and culturing conditions

Standard conditions were used for *C. elegans* cultivation at 20 °C [28]. Nematode growth medium (NGM) was prepared following the standard protocol, except that casein digest (3 g/L) was used instead of peptone. Animals were cultured on NGM agar plates seeded with *Escherichia coli* strain OP50 bacteria for feeding [29]. Animals were age-synchronized by hypochlorite bleaching [28] and hatched overnight in M9 buffer at 20 °C to obtain synchronized larval stage (L1) worms. *C. elegans OW40 (zgls15[P(unc-54)::αsyn::YFP]IV)* strain, from Van Ham et al. 2008, was used for the α-syn inclusions and motility experiments [30]. This strain overexpresses the wild-type human α-syn fused to the Yellow Fluorescent Protein (YFP) under the control of the muscle-specific promoter, *unc-54*. Muscle expression was chosen to allow for efficient RNAi and easy visual detection of inclusions. *C. elegans* N2 (wild-type) strain was obtained from the Caenorhabditis Genetics Center (CGC) (https://cgc.umn.edu).

### RNAi experiments

For RNAi experiments, synchronized L1 worms were grown on RNAi plates (NGM plates supplemented with 12.5 mg/mL isopropyl β-D-thiogalactoside (IPTG) and 50 mg/mL ampicillin), seeded with *Escherichia coli* strain HT115 expressing specific dsRNA against the genes of interest. The following RNAi strains were tested: empty vector (L4440, control), *tdo-2*, and *ftn-1* from the Ahringer bacterial library [31] or SNCA RNAi designed in-house against the CDS of human α-syn. At the fourth larval stage (L4), 2.5 mg/L 5-Fluoro-2’-deoxyuridine (FUDR) (Sigma, F0503-1G) was added to the plates to inhibit the growth of offspring. Worms were cultivated with the RNAi bacteria until day 4 of adulthood (D4, 4 days after the L4 stage) and collected for subsequent imaging or locomotion experiments.

### Quantitative RT-PCR

To assess the efficiency of RNAi knockdown, total RNA was extracted from RNAi-treated worms using TRIzol reagent (#15596018, Invitrogen) according to the manufacturer’s protocol. The RNA concentration and quality were measured with a NanoDrop 2000 spectrophotometer. After this, cDNA was synthesized with the RevertAid H Minus First Strand cDNA Synthesis kit (#K1632, Life Technologies) using random hexamer primers. Quantitative real-time PCR was performed using a Roche LightCycler 480 Instrument II (Roche Diagnostics) with SYBR green dye (#172-5125, Bio-Rad) to detect cDNA amplification. Relative transcript levels were quantified using a standard curve of pooled cDNA samples. Expression levels were normalized against *pmp-3,* an endogenous reference gene (Supplementary Table 5).

### Pharmacological assays with Fer-1 and DFO in C. elegans

Fer-1 and DFO plates were made by adding the drugs into the NGM after autoclaving, giving a final concentration of 240 μM for Fer-1 and 100 μM for DFO. DMSO was used as the vehicle. Synchronized L1 worms were grown on regular NGM agar plates seeded with *E. coli* OP50 bacteria until the L4 stage and then transferred to Fer-1, DFO, or DMSO-containing plates with FuDR. Worms were treated with the drugs or vehicle for 4 days, until day 4 of adulthood, and were collected for subsequent imaging or locomotion experiments.

### Quantification of α-inclusions

*C. elegans* OW40 day worms were mounted on 3% agarose pad and anesthetized using 25 mM Levamisole (Sigma). Z-stack images of the head region of the worm were obtained by using a Zeiss Axio Imager 2 microscope and a ×40 oil objective. Using ImageJ and FIJI, a maximum intensity projection of the z-stack was obtained, the background was subtracted (rolling ball radius of 10 pixels), and a threshold (automatic) was applied to the image. The number of fluorescent spots >1 μm^2^ in the area between the tip of the head and the end of the last pharyngeal bulb was quantified, assisted by the Fiji analyze particle function, and manually curated for overlapping inclusions [32]. The experiments were performed in triplicate, and at least 10 worms were quantified for each condition.

### Locomotion analysis (thrashing)

Wild-type or OW40 worms were washed twice with M9 to remove bacteria and transferred to an empty NGM agar plate for tracking. The plate was flooded with M9, and the thrashing (swimming) frequency was acquired after recording the moving animals for 30 s at 20 frames per second. The WF-NTP software was used to analyze the movies and derive the thrashing frequency (number of bends/30 s) [33]. Each experiment was performed in triplicate.

### Iron measurement in *C.elegans*

Ten technical replicates of each genotype (200 individual *C. elegans* per replicate), were washed multiple times and lyophilized overnight. All 40 samples (10 replicates/genotype × 4 genotypes) were digested in 20 µL of 65% HNO3 for 15 h at room temperature (around 22 °C), heated up at 90 °C for 20 min, then diluted to 200 µL using 1% HNO3. Six matrix blanks (no *C. elegans*) were processed to quantify the background metal levels. Inductively coupled plasma-mass spectrometry: Agilent Technologies 8800x ICP-MS was operated using a previously published method [34].

### Statistical analysis

Statistical analyses of the human data were carried out on GraphPad Prism 8.4.2 (GraphPad Software, USA) for Windows. All raw datasets were run through diagnostics prior to performing any analyses, to check for parametric assumptions; the tests used in each analysis were chosen accordingly. Furthermore, outliers were checked and removed when significant. A one-way analysis of variance (ANOVA) with Sidak post-hoc test or non-parametric Kruskal–Wallis with Dunn’s post-hoc test was done to compare ferroptosis-related markers’ expression between α-syn pathology, as well as differences between the disease conditions and healthy controls, and differences within anatomical subregions of the SN within controls. Due to the number and distribution of our cell culture data, a non-parametric Kruskal–Wallis test with Dunn’s post-hoc comparison was used to determine the statistical significance of the treatment effects. Graphs were plotted as violin plots (with medians). Furthermore, non-parametric Spearman correlation analyses were used to determine whether there was a positive or negative correlation between each ferroptosis marker’s expression and the naturally occurring neuromelanin in the dopaminergic neurons. A further simple linear regression analysis was done to determine the statistical significance of the correlations performed for each marker. Graphs were plotted as scatter plots with a line of best fit ±95% confidence intervals. All results were considered statistically significant at *p* < 0.05. Furthermore, Spearman’s correlation test and linear regression analysis were performed to assess the multivariate effects concerning the immunohistochemistry post-mortem human data, as well as an MLR using IBM SPSS (28.0.1.0) to assess the influence of these variables on the pixel density scoring and DSP outputs. For the analysis of PI incorporation into cells, normal distribution was tested, and significance was analyzed using ANOVA multiple comparisons. Cell viability was represented as mean ± standard error of the mean (SEM). Statistical significance was defined as ^*^p < 0.05. All experiments were repeated three times, and data were collected and analyzed in an unbiased manner. Graphs and statistical analysis of results from *C. elegans* experiments were performed using GraphPad Prism 10. Data shown are presented as scatter or mean ± SEM. Statistical significance was calculated by one-way ANOVA and Dunnett’s multiple comparisons post-hoc test, with *p* < 0.05 considered statistically significant. Statistical significance was denoted as follows: ^****^p < 0.0001; ^***^p < 0.001; ^**^p < 0.01 and ^*^p < 0.05.

## Results

### α-syn pathology in the PD brain is linked to ferroptosis-related markers

To establish the link between α-syn pathology and ferroptosis, we analyzed the expression of ferroptosis-related markers in dopaminergic cells in the brain of age-matched controls, and compared them to α-syn pathology-positive cells from the PD patient group (Fig. 1). We focused on ferroportin, ferritin, NCOA4, cytochrome c, GPX4, and 4HNE. Ferritin expression was lower in dopaminergic cells containing α-syn, compared to dopaminergic cells from age-matched controls (one-way ANOVA, Sidak, *p* = 0.0015). Ferroportin showed a significant decrease in cells with (and without) α-syn, compared to cells from age-matched controls (Kruskal–Wallis, Dunn, *p* < 0.0001 and *p* = 0.0213, respectively). An increasing trend was observed with 4HNE in dopaminergic cells containing α-syn, compared to cells from age-matched controls; however, this was not significant, while there was a significant increase in 4HNE in cells without α-syn, compared to cells from age-matched controls (one-way ANOVA, Sidak, *p* = 0.0172). Finally, NCOA4, GPX4, and cytochrome c did not show any significant change between any of the tested groups. To investigate if the expression of the markers was also affected by the presence of the pathology itself, we compared the expression of these proteins between α-syn-positive and α-syn-negative cells within the same diseased PD brains. Ferritin was decreased in α-syn-positive cells, compared to α-syn-negative cells from PD patients (one-way ANOVA, Sidak, *p* = 0.0231). Similarly, ferroportin was decreased in α-syn positive cells, compared to α-syn negative cells from PD patients (one-way ANOVA, Sidak, *p* = 0.0132). No other significant change was observed when comparing pathology-positive and pathology-negative cells (Fig. 1B). Moreover, we found no strong correlation between the levels of neuromelanin, an effective metal chelator, and protein levels of ferroptosis-related markers (simple linear regression *r*^2^ = 0.0007-0.2529, *p* < 0.05) which excludes the possible effect that neuromelanin could have had on the positive pixel density scoring in SN (Fig. 1C). Next, we analyzed whether differences in age, post mortem delay (PMD) and fixation time (FT) between control and PD patients could have had an effect on the above-mentioned results. Overall, the correlations between pixel density scores, age, PMD and FT were very weak and not significant (Spearman’s correlation test and simple linear regression, *r*^2^ = 0.0003–0.2648, *p* = 0.0170-0.9453) (Supplementary Fig. 1). The multiple linear regression (MLR) analysis shows that donor’s age and sex, as well as post-mortem delay and fixation times did not elicit a significant effect on the reported pixel density scoring.

Across all markers, the addition of these variables to the model, explained a maximum of 18.5% of variability in pixel density scoring values (Supplementary Fig. 2A). Moreover, where the variability was significantly increased, the change was deemed negligible, with the highest variability change reported at 10.8% in ferroportin, while all other markers did not show an R-squared change of >7.1% (significant or not) (Supplementary Fig. 2A). This indicates that the significant changes observed in positive pixel density scores across all analyzed ferroptotic-related proteins is indeed a result of the protein’s expression and unlikely to be affected by individuals’ age, sex, FFPE age, post-mortem delay or fixation time. These findings suggest a potential link between α-syn pathology and ferroptosis in Parkinson’s disease, particularly through the dysregulation of iron homeostasis markers, including ferritin and ferroportin, which may contribute to the vulnerability of dopaminergic neurons.

### SNc subregions are differentially susceptible to ferroptosis

To investigate the distribution of ferroptosis-related marker expression within the SNc, this area was divided into three subregions: the lateral, ventral, and medial SNc according to their associated functions and connectivity (Fig. 2A). All markers, with the exception of GPX4 (one-way ANOVA, Sidak, *p* > 0.05), were altered between SNc sub-areas. Ferritin and cytochrome c expression was significantly higher in ventral (Kruskal–Wallis, Dunn, *p* = 0.0007 and *p* = 0.0009, respectively) and medial areas (Kruskal–Wallis, Dunn, *p* < 0.0001 in both markers) compared to the lateral SNc. However, this change was more nuanced in cytochrome c. Ferroportin, 4HNE, and NCOA4 showed a higher marker expression in medial SNc compared to ventral (Kruskal–Wallis, Dunn, *p* = 0.0388, *p* < 0.0001, and *p* = 0.0011, respectively) and lateral (Kruskal–Wallis, Dunn, *p* < 0.0001, in the three markers).

Overall, except for GPX4, all markers had higher expression in the medial SNc compared to lateral and/or ventral SNc, while significant changes between ventral and lateral SNc were observed in ferritin and cytochrome c only.

### Digital spatial profiling of PD brains reveals significantly differentially expressed genes related to the ferroptosis pathway

To further investigate the alterations to the ferroptotic pathway observed at the protein level, particularly in iron-handling markers including ferritin and ferroportin, we extended our analysis to the transcriptomic level. Since differences were not consistently detected across all six proteins examined, and because protein and transcript levels are not always directly correlated, we used gene expression profiling to capture broader ferroptosis pathway changes that may not be reflected at the protein level alone.

**Fig. 3:**
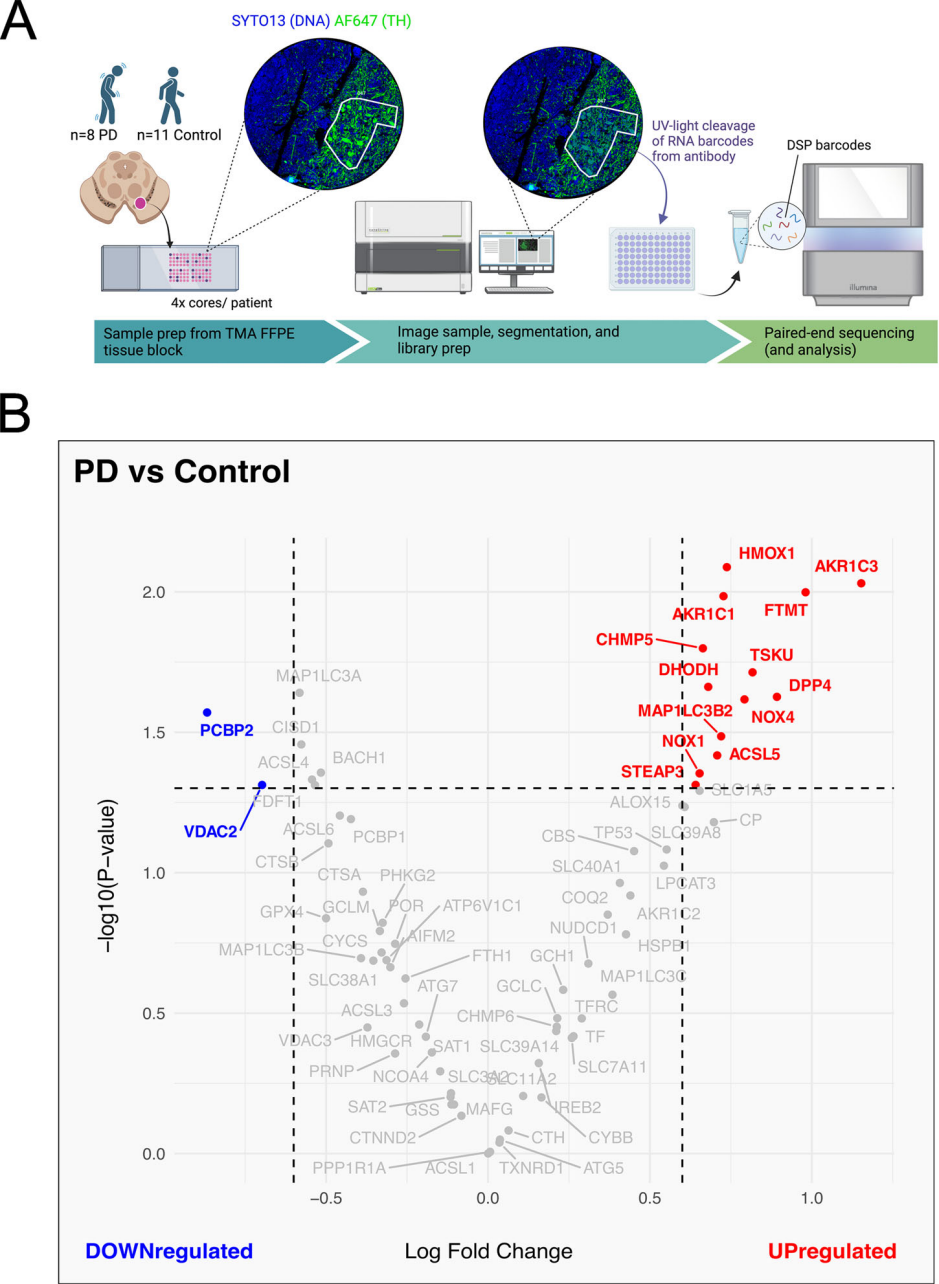
Ferroptosis-related transcriptomic changes in Parkinson’s disease (PD) patients and age-matched controls. **A** Summary experimental layout of the digital spatial profiling (DSP) protocol, using a tissue microarray (TMA) slide including ~ 4 cores/ patient from the substantia nigra of PD (n = 8) and age-matched controls (*n* = 11). An example core is shown, fluorescently labeled with anti-tyrosine hydroxylase (AF647 TH) (Alexa Fluor® 647-conjugated, green), for targeted dopaminergic cell isolation, and SYTO13 as a nuclear/DNA label (blue). ROI selection(s) were then imaged, and probes with barcodes from TH-positive areas were then cleaved off the antibody with ultraviolet (UV) light. RNA barcodes were collected for sequencing. **B** Volcano plot showing the results of the DSP of ferroptosis-related transcripts. Limma analysis revealed 13 ferroptosis-related upregulated differentially expressed genes (DEGs) (red) and 2 downregulated ferroptosis-related DEGs (blue) in PD dopaminergic neurons, compared to dopaminergic neurons from age-matched controls (LogFC2 > 0.6, adjusted *p* < 0.05). Graphics adapted from GeoMx® Nanostring, created using Biorender.com.

We performed digital spatial profiling (DSP) in the SN of PD patients versus healthy age-matched controls (Fig. 3A). Out of the 76 ferroptosis-related genes analyzed, we revealed 13 to be significantly upregulated and 2 significantly downregulated in PD brains compared to controls (Fig. 3B). These global changes suggest a shift towards favoring ferroptosis in the α-syn-related pathology of PD. The Multiple Linear Regression (MLR) analysis shows that TH-positive area explains 70.5% (*p* < 0.001) of the variability in unique mRNA counts from PD and control patients (Supplementary Fig. 2B). The age of the FFPE block slightly increases the adjusted R-squared to 0.722 (*p* = 0.036), and fixation time raises it to 0.743 (*p* < 0.012), adding only 3.7% more explained variability. Post-mortem delay has no significant impact (*p* = 0.998) (Supplementary Fig. 2B). Thus, unique mRNA counts are mainly influenced by the TH-positive area, not by tissue quality variables, suggesting that the presence of α-syn pathology in dopaminergic neurons from PD patients is linked to altered gene expression of ferroptosis-related genes.

Ferritin (FTH1) and ferroportin (SLC40A1) were not significantly altered at the transcript level in PD samples (Fig. 3B, in gray, and Supplementary Table 6). Despite the lack of statistical significance, these transcript-level patterns may still reflect underlying regulatory shifts, especially considering the significant decrease observed in ferritin and ferroportin at the protein level. This suggests that biologically meaningful changes may be present even when not captured as significant at the transcriptomic level, particularly for genes subject to complex post-transcriptional and translational regulation.

### RSL3 induced PI uptake in the dopaminergic neurons

To investigate whether challenge with α-syn fibrils could increase neuronal vulnerability to ferroptosis, we first selected RSL3 as a model compound for the induction of ferroptosis. RSL3 has been extensively used as a ferroptosis inducer [16, 27, 35]. The compound is a powerful inhibitor of the enzyme GPX4, an essential player in the cellular defense against lipid peroxidation and the maintenance of optimal cellular redox balance [36].

First, we treated PCNs and LUHMES cells with RSL3 and evaluated which concentration of RSL3 would not induce increase in PI uptake and thus cell death (Supplementary Fig. 4A). We observed no cell death at 100 nM RSL3, as reflected by PI uptake (Supplementary Fig. 4B). The aim of this experiment, however, was not to identify concentrations that cause significant toxicity, but rather to establish RSL3 doses that do not induce significant cell death, to serve as a sensitizing background for α-synuclein co-treatment. In LUHMES cells, concentrations of 5 nM, 10 nM, and 50 nM were initially tested, as prior studies indicated that these cells are particularly susceptible to ferroptosis [37]. This prompted us to test lower concentrations of RSL3, including 100, 500, and 1000 pM, and we did not observe cell death, as measured by PI-positive cells (Supplementary Fig. 4C), with 1000 pM being the highest concentration that did not significantly increase PI uptake. Given LUHMES cells’ higher vulnerability to ferroptosis, we further tested whether ferrostatin-1 (Fer-1), a known ferroptosis inhibitor, could rescue cells from RSL3-induced ferroptotic death. Fer-1 successfully prevented the RSL3-induced increase in PI uptake in all tested conditions, even at higher concentrations of RSL3 (Supplementary Fig. 4D). These results demonstrate that dopaminergic LUHMES cells are more susceptible to ferroptosis compared to PCN neuronal cells.

### α-syn fibrils induce PI uptake in PCNs and LUHMES in a concentration-dependent manner

**Fig. 4:**
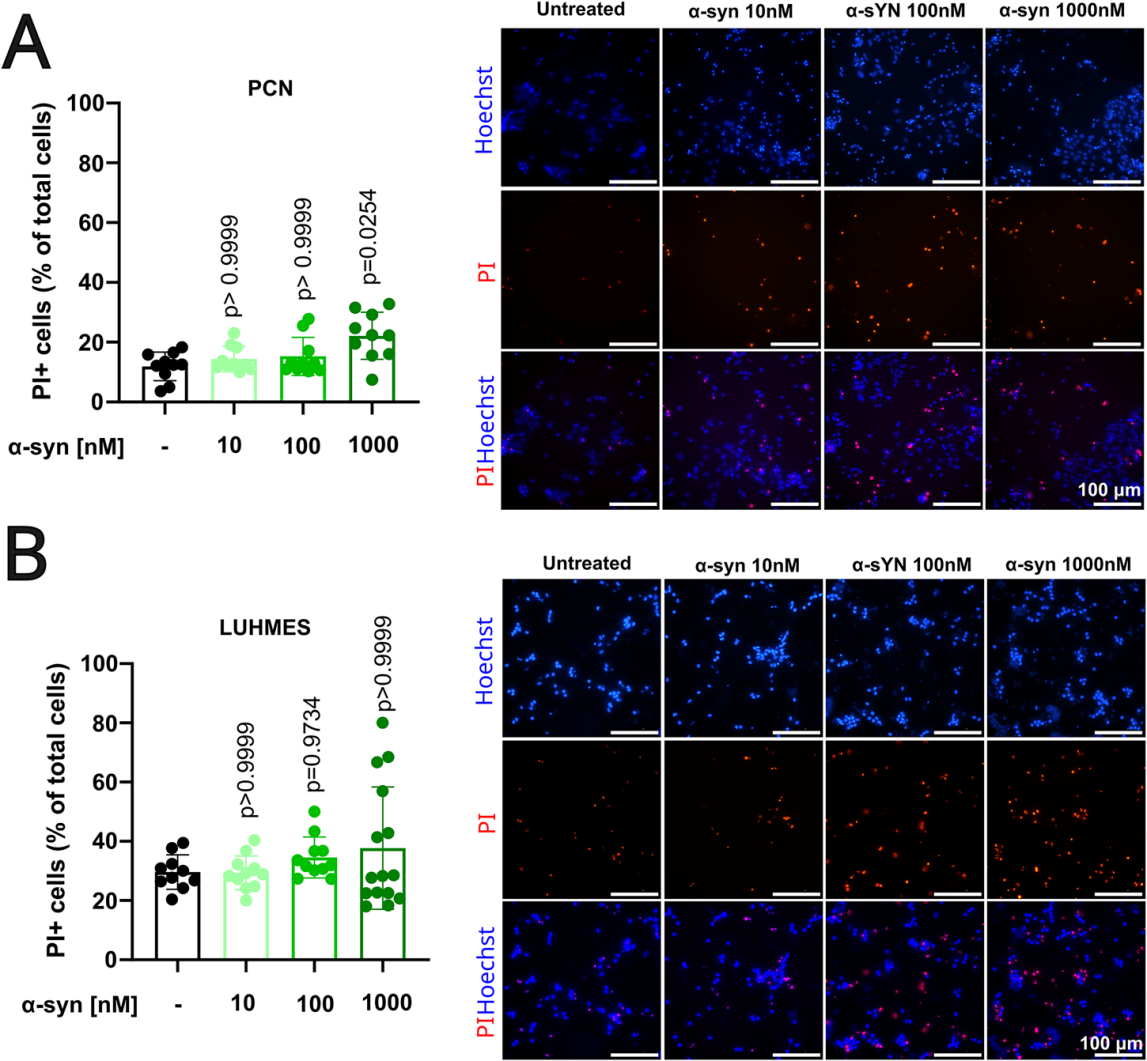
PI uptake in PCNs and LUHMES treated with α-syn fibrils. **A** Quantification of PI-positive cells in relation to total cells showed an PI uptake with higher α-syn concentration in PCNs (left) and the representative images after 24 h treatments (right). **B** Quantification of PI-positive cells in differentiated LUHMES cells (left) and the representative images after α-syn 24 h treatments (right). Each graph in this figure is a representative experiment with 10–15 technical replicates from a total of three biological replicates. Each experiment was also conducted independently to ensure the robustness and consistency of our results and similar trends were observed in all biological replicates. Statistical significance was determined by Kruskal–Wallis with Dunn’s post-hoc multiple comparison test (*p* < 0.05).

We next established the response of PCNs and LUHMES dopaminergic cells to α-syn fibrils at various concentrations (10, 100, and 1000 nM) for 24 h, and analyzed for the PI uptake. Both PCNs (Fig. 4A) and LUHMES dopaminergic neurons (Fig. 4B) showed a mild increase in PI uptake in response to α-synuclein fibrils. Based on these results, we selected 100 nM for PCNs and 1 µM for LUHMES neurons for subsequent experiments.

These concentrations produced relatively mild but noticeable effects on PI uptake, enabling us to explore potential synergistic interactions with ferroptotic stressors. Although α-synuclein fibrils alone caused a statistically significant increase in PCN in PI uptake, the effect remained limited, supporting their use in combination studies.

### Combination of α-syn fibrils and RSL3 increases PI uptake in both PCNs and LUHMES dopaminergic neurons

**Fig. 5:**
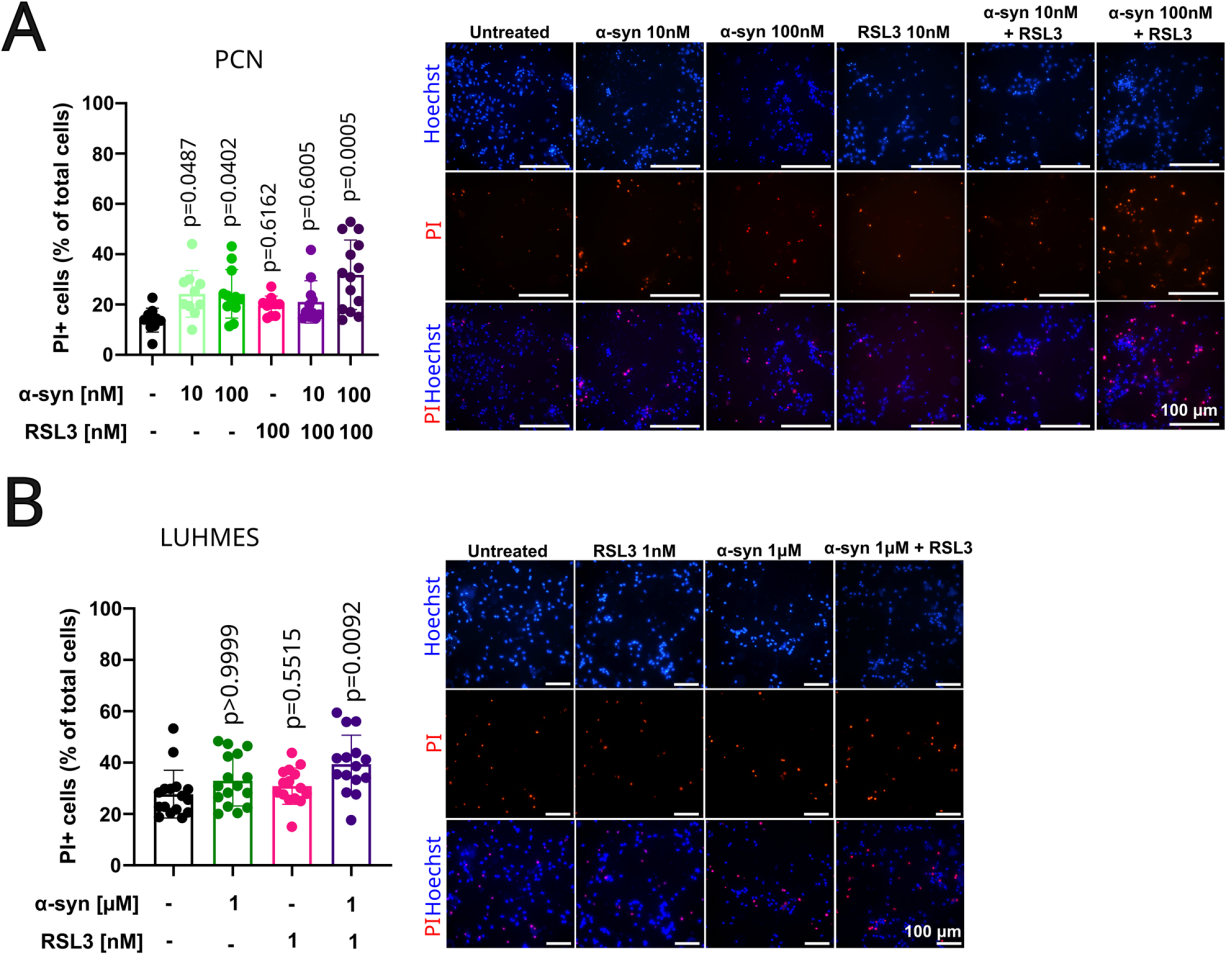
Combined challenge of α-syn fibrils with RSL3 induced higher PI uptake in neuronal cells. **A** Quantification of PI-positive cells in relation to total cells showing the levels of PI uptake with RSL3 and α-syn treatments alone compared to higher PI positivity with co-treatment of 100 nM α-syn fibrils in PCNs (left) and the representative images after 24 h treatments (right). **B** Quantification shows no significant PI uptake with RSL3 and α-syn treatments alone and a higher PI positivity with co-treatment of RSL3 and 1 µM α-syn fibrils in LUHMES (left) and the representative images after 24 h treatments (right). Each graph in this figure is a representative experiment from a total of three biological replicates. Each replicate was conducted independently to ensure the robustness and consistency of our results, and similar trends were observed in the remaining two replicates.) Statistical significance was determined by Kruskal–Wallis with Dunn’s post-hoc multiple comparison test (*p* < 0.05).

Building on our findings regarding PI uptake responses to individual RSL3 and α-syn fibril challenge, we further explored whether α-syn can increase ferroptotic cell death when a ferroptotic stimulus is also present. Therefore, we examined their combined impact on PI uptake, regarded as a proxy for cell death measurement, by co-treating PCNs and LUHMES dopaminergic cells with low concentrations of RSL3 and α-syn. We next challenged PCNs with 100 nM RSL3 and combined it with either 10 nM or 100 nM α-syn (Fig. 5A). In primary cortical neurons (PCNs), treatment with 100 nM α-synuclein alone led to a 63.95% increase (median: 22.51 vs 13.73) in PI uptake compared to untreated cells (*p* = 0.0402), while 100 nM RSL3 alone resulted in a smaller, non-significant increase of 46.61% (median: 20.13 vs. 13.73) (*p* = 0.6162). However, the co-treatment with both 100 nM α-synuclein and 100 nM RSL3 caused a markedly higher increase in PI uptake, being 131.03% (median: 31.72 vs. 13.73) compared to untreated cells (*p* = 0.0005). A similar trend was observed in LUHMES dopaminergic neurons, where 1 µM α-synuclein alone increased PI uptake by only 14.59% (median: 30.16 vs. 26.32) (*p* > 0.999), and 1 nM RSL3 alone by 17.59% (median: 30.95 vs 26.32) (*p* = 0.5515). Yet, the combination of 1 nM RSL3 and 1 µM α-synuclein led to a significantly greater PI uptake, with an increase of 46.66% (median: 38.6 vs. 26.32) (*p* = 0.0092) compared to untreated cells (Fig. 5B). In summary, these results suggested an increased vulnerability of PCNs and LUHMES cells to ferroptosis when α-syn fibrils and ferroptosis inducer are present concomitantly in low concentration.

### Ferroptosis inhibitors prevent the cell death mediated by α-syn and RSL3 co-treatment

To further evaluate whether targeting the ferroptosis pathway could protect against α-syn-induced cell death, we tested the effects of Fer-1 and DFO on PI uptake. While Fer-1 scavenges and neutralizes ROS and lipid radicals, DFO inhibits ferroptosis by sequestering iron ions, thereby restricting their ability to catalyze the Fenton reaction and reducing ROS production.

**Fig. 6:**
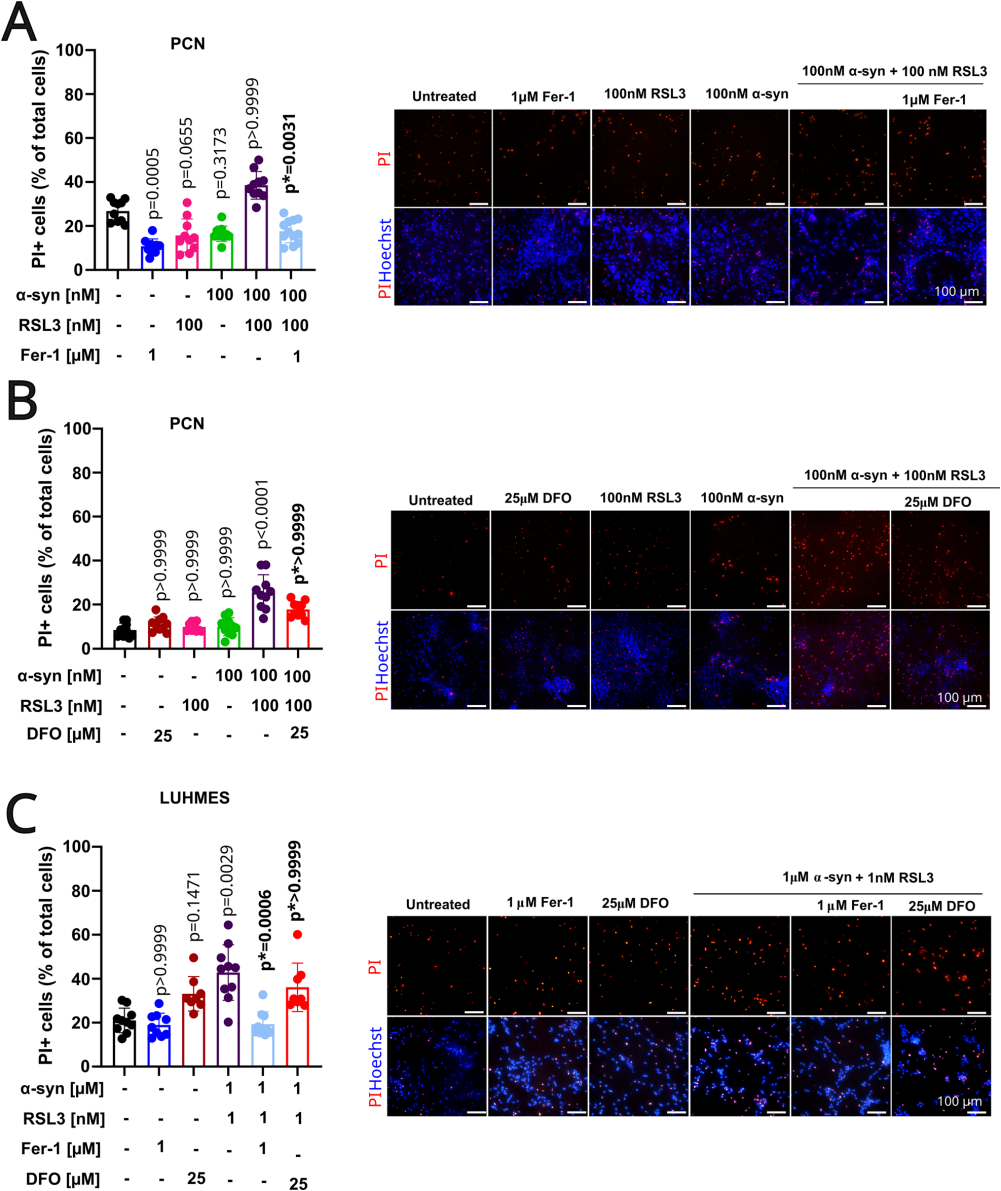
The cell death mediated by α-syn fibrils and RSL3 co-treatment was differentially reduced by Fer-1. **A** PCNs were pre-treated for 4 h with 1 µM Fer-1 followed by a 24 h treatment with RSL3, α-syn, or both, and Fer-1. The quantification shows that PI-positive cells in conditions of co-treatment of α-syn and RSL3 were reduced by Fer-1 (left, *p* = 0.0031). Representative images following the treatment are presented on the right side of the panel. **B** PCNs were pre-treated for 4 h with 25 µM DFO, followed by a 24 h treatment with RSL3, α-syn, or both and DFO. The quantification shows that PI-positive cells of co-treatment of α-syn and RSL3 were not significantly decreased by DFO (left, *p* > 0.999). Representative images following treatment are shown on the right side of the panel. **C** LUHMES were pre-treated for 4 h with 1 µM Fer-1 or 25 µM DFO, followed by a 24 h treatment with RSL3, α-syn, or both, and Fer-1 or DFO. Fer-1 (*p* = 0.0006) but not DFO (*p* > 0.9999) reduced the PI-positive cells in conditions of RSL3 and α-syn, as shown by the image quantification. The representative images after the treatment are presented on the right side of the panel. Each graph in this figure is a representative experiment from a total of three biological replicates. Each replicate was conducted independently to ensure the robustness and consistency of our results, and similar trends were observed in all biological replicates. Statistical significance was determined by Kruskal–Wallis with Dunn’s post-hoc multiple comparison test (*p* < 0.05; *p*, compared to untreated conditions; p^*^, compared to co-treatment conditions).

Our results indicate that Fer-1 significantly protects against cell death induced by co-treatment with α-syn fibrils and RSL3 in PCNs, showing a 55.7% reduction (median: 16.81 vs. 37.93) compared to the α-syn and RSL3 group (*p* = 0.0031; Fig. 6A). In contrast, DFO did not provide significant protection (*p* > 0.9999), despite a 29.3% reduction (median: 17.73 vs. 25.07) in cell death (Fig. 6B)*.*

In human dopaminergic LUHMES cells, Fer-1 also prevented co-treatment-induced cell death, resulting in a 62.5% reduction (median: 16.79 vs. 44.72) (*p* = 0.0006; Fig. 6C). However, DFO again failed to show significant neuroprotection (p > 0.9999), with only a 31.0% reduction (median: 30.85 vs. 44.72) compared to the α-syn and RSL3 condition (Fig. 6C).

### Ferroptosis inhibition reduces α-syn inclusions in a PD C. elegans model

**Fig. 7:**
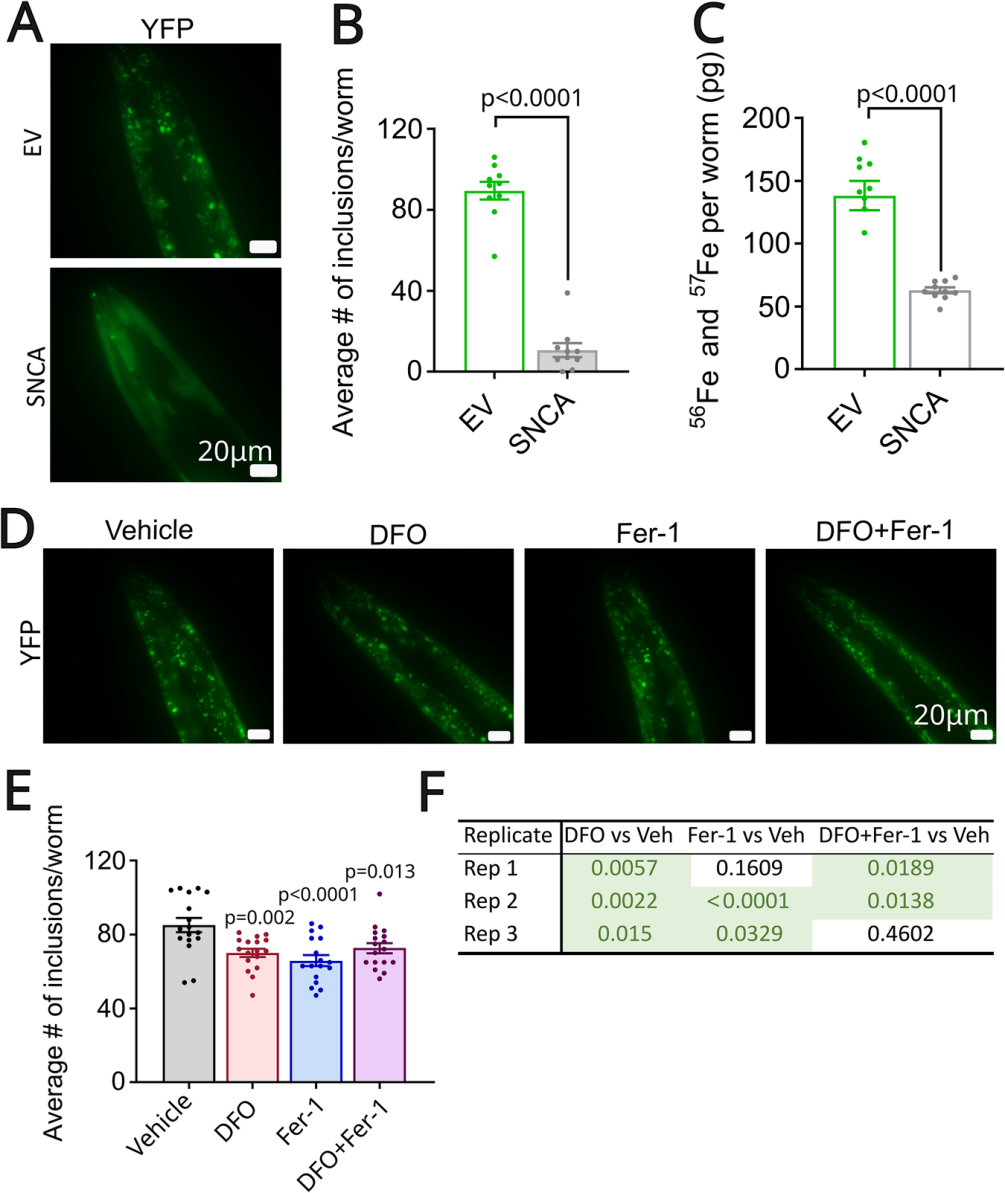
Ferroptosis inhibition reduces the level of α-syn inclusions that are linked to iron accumulation in *C. elegans*. **A** Representative fluorescent (YFP) images of α-syn inclusions (foci) in the head of α-syn overexpressing worms (empty vector = EV) compared to worms where α-syn was knock-down by RNAi against α-syn (SNCA RNAi). **B** Quantification of α-syn inclusions larger than 1 μm2 in the head region per animal; *n* = 10 worms per condition/experiment. One representative experiment is shown. Higher levels of α-syn inclusions can be observed in α-syn overexpressing worms (EV) compared to worms where α-syn was knock-down by RNAi against α-syn (SNCA). **C** Levels of total iron (Fe) are significantly increased in control worms compared to SNCA RNAi worms, ^****^p < 0.0001; *n* = 10 (200 individual *C. elegans* per replicate). One representative experiment is shown. **D** Representative fluorescent (YFP) images of α-syn inclusions (foci) in the head of day 4 adult worms treated with vehicle (DMSO 2%), deferoxamine (DFO, 100 μM), ferrostatin-1 (Fer-1, 240 μM), and a combination of DFO and Fer-1. **E** Quantification of α-syn inclusions larger than 1 μm^2^ in the head region per animal, ^**^p = 0.002, ^****^p < 0.0001, ^*^p = 0.013; *n* = 17 worms per condition. One representative experiment out of 3 is shown. **F** *p*-values from the three independent replicates, of which Replicate number 2 is shown in (**E**). Statistical significance was determined by an unpaired *t*-test or one-way ANOVA with multiple comparisons (*p* < 0.05).

To further confirm our hypothesis regarding the involvement of ferroptosis in PD pathology, we utilized an α-synucleinopathy animal model, a *C. elegans* system expressing human α-syn fused with yellow fluorescent protein (YFP) in the body-wall muscle cells [30]. On the regular *C. elegans* laboratory diet, α-syn forms inclusions that can be easily visualized and studied by fluorescence microscopy throughout the lifespan of these animals. The inclusions become aggregates with age [30], resembling the pathology found in PD patients [38, 39]. The presence of α-syn causes cellular toxicity, leading to an accelerated age-related decline in the motility of the worms. To establish a possible link between ferroptosis and α-syn, we first tested whether α-syn influenced the iron levels in the worms by comparing α-syn worms with and without RNAi knockdown of α-syn. Indeed, knockdown of α-syn not only reduced the number of inclusions in the worms but also lowered the iron levels by two-fold (Fig. 7A–C). Other metals, such as Manganese (Mn) and Zinc (Zn), were also significantly downregulated by RNAi of α-syn, while no alterations were observed in Copper (Cu) levels (Supplementary Fig. 5A, B). Given that ferritin expression is lower in α-syn-positive dopaminergic neurons compared to α-syn-negative neurons in PD brains, we evaluated the impact of ferritin loss by knocking down its ortholog *ftn-1* in α-syn overexpressing worms and studying α-syn inclusions and motility. Reducing the expression of ferritin, a ferroptosis-related gene, which is responsible for iron storage and prevention of the accumulation of free iron in the cell, may worsen the toxicity induced by α-syn overexpression, but has not been reproduced in three independent experiments (Supplementary Fig. 5C).

To determine whether ferroptosis inhibitors targeting different parts of the ferroptotic pathway could influence the toxicity of α-syn, we treated the worms with DFO and Fer-1 (separately and in combination). Iron chelation with DFO was the only treatment that consistently reduced the number of inclusions across all the experiments. Fer-1 and the combination of DFO and Fer-1 also decreased the number of α-syn inclusions, but only in two out of three independent experiments (Fig. 7D–F*,* Supplementary Fig. 5E). However, the combination of DFO and Fer-1 decreased the number of α-syn inclusions, although this reduction was not linked to improved motility in the worms (Supplementary Fig. 5F). Altogether, these data suggest that iron accumulation and lipid peroxidation, both inducers of ferroptosis, can enhance inclusion formation of α-syn. However, whether inhibition of these ferroptosis-related processes and the associated reduction in inclusions protects against α-syn-induced loss of motility at more advanced ages remains to be established.

## Discussion

**Fig. 8:**
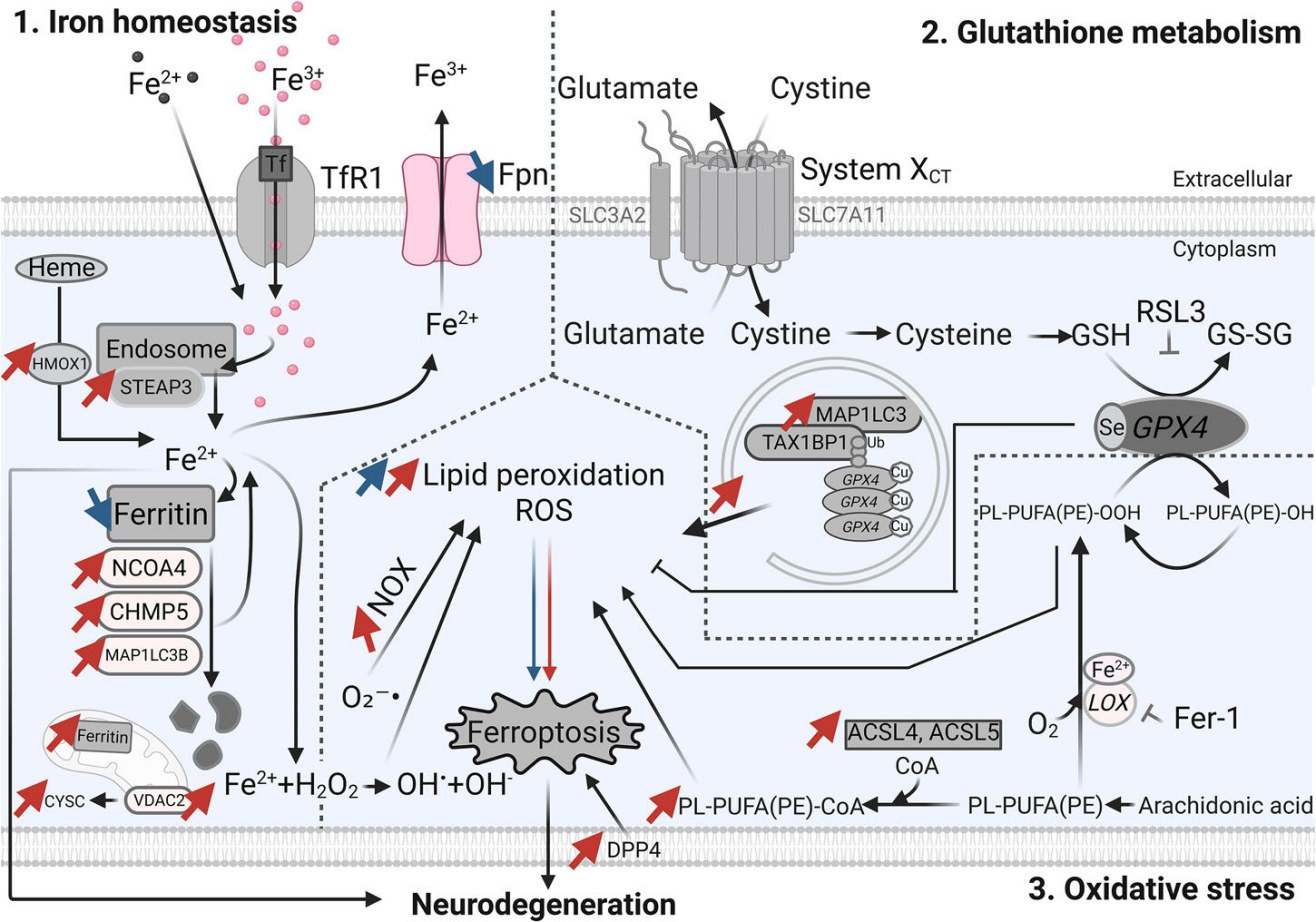
Changes on the protein (blue arrows) and transcriptomic level (red arrows) affecting the cellular mechanism related to the ferroptotic pathway. Iron metabolism (left), cysteine and glutathione metabolism (top right), and polyunsaturated fatty acid metabolism (bottom right). ACSL4; Long-chain-fatty acid—CoA ligase 4, ACSL5; acyl-CoA synthetase long chain family member 5, AKR1C1; aldo-keto reductase family 1 member C1, AKR1C3; aldo-keto reductase family 1 member C3, CHMP5; charged multivesicular body protein 5, Cu; Copper, CYCS; cytochrome c, DHODH; dihydroorotate dehydrogenase, mitochondrial enzyme, DPP4; dipeptidyl peptidase 4, Fe^2+^; ferrous iron, Fe^3+^; Ferric iron, Fpn; Ferroportin, GPX4; glutathione peroxidase 4, GSH; glutathione (reduced glutathione form), GS-SG; glutathione disulfide (oxidized glutathione form), HMOX1; heme oxygenase 1, LOX; lipoxygenase, MAP1LC3; microtubule-associated protein 1 light chain 3, NCOA4; nuclear receptor co-activator 4, NOX; NADPH oxidase 4, PL-PUFA(PE)-OH; non-toxic polyunsaturated fatty-acids, PL-PUFA(PE)-OOH polyunsaturated fatty acid-containing-phospholipid hydroperoxides, ROS; reactive oxygen species, RSL3 (1S,3R)-RSL3, RTAs radical-trapping antioxidants, Se; Selenocysteine, SLC3A2; solute carrier family 3 member 2, SLC39A14; solute carrier family 39 member 14, SLC7A11; solute carrier family 7 member 11, STEAP3; metalloreductase. System XcT; glutamate/cystine antiporter system, TAX1BP1; Tax1 Binding Protein 1, TfR1; transferrin 1 receptor, TSKU; tsukushi, small leucine-rich proteoglycan, Ub; ubiquitin, VDAC2; voltage-dependent anion channel 2. Parts of the figure were made with the BioRender program.

This study suggests that the ferroptotic pathway contributes to PD pathology, particularly in relation to α-syn toxicity and aggregation. Ferroptosis-related markers have been studied in relation to α-syn using several models of PD [40–43]. However, in this study, we investigated for the first time a group of specific markers of ferroptosis in relation to α-syn inclusions in human post-mortem brains of PD patients and corresponding age-matched controls, both at the protein and transcriptomic level (Figs. 1 and 3). The combined results from these two approaches reveal increased vulnerability to ferroptosis linked to PD pathology (Fig. 8*,* Supplementary Table 7).

Consistent with the current literature supporting the crucial link between ferritin and neurodegenerative diseases, α-syn was associated with a significant decrease in both ferritin and ferroportin expression, compared to cells from age-matched controls and/or pathology-negative cells from PD patients [40, 41, 43, 44]. Furthermore, 4HNE levels in cells with α-syn align with previous research indicating that heightened oxidative stress in nigral neurons may contribute to cell death in PD [45, 46]. This is expected, as 4HNE is a by-product of lipid peroxidation, which precedes ferroptotic cell death. Surprisingly, NCOA4 did not show any significant changes in its expression in the presence of α-syn inclusions in the human PD brain. This was unexpected, given the decrease in ferritin expression and recent study reporting increased NCOA4-mediated ferritinophagy in PD mice [47]. This could be attributed to the decrease in ferritin level, associated with an increase in redox-active iron seen in PD patients with extensive neurodegeneration, as well as an increased formation of ROS and iron accumulation [48], which may be influenced by factors independent of NCOA4-mediated ferritinophagy. Furthermore, the relative function of NCOA4-mediated ferritinophagy remains largely unexplained due to a lack of neurodegenerative and ageing pathological models to study its effects. The iron status and the consequent results on NCOA4 are highly variable and largely dependent on the model used [49] or potentially species-specific differences.

Despite being the most representative model of PD, investigating the post-mortem human brain is limited by a variety of factors, making interpretation of the results challenging. It is unclear how far the ferroptotic pathway affected neuronal cells, potentially explaining why changes in the rest of the analyzed markers are not seen. This limitation may also apply to the undetected changes in GPX4 levels. However, GPX4 has also been shown to co-localize with neuromelanin and be up-regulated in the SN of PD brains [42]. Since neuromelanin is normally present in neurons that have not yet died, it is speculated that neuromelanin, known to function as an iron chelator [50], may protect the remaining neurons from ferroptotic cell death. However, no significant correlation with neuromelanin was observed in this study (Fig. 1C). Finally, cytochrome c did not show any changes in expression in comparison to cells from age-matched controls, nor from pathology-negative cells from PD patients. Existing literature has shown cytochrome c to be upregulated in cell culture experiments used as PD models [51]. However, cytochrome c is a marker of apoptosis, as well as a product released from mitochondrial dysfunction and increased oxidative stress, and not solely a ferroptosis-specific marker [14]. The conditions that precede mitophagy are characteristic of normal aging. Therefore, the changes observed in cells with α-syn pathology may also occur in cells from age-matched controls and in pathology-negative cells within the PD group. Moreover, some cells in the PD patient tissue contained more advanced α-syn aggregates (Lewy bodies), while others had only diffuse forms of α-syn, which are indicative of different stages of PD [52], and may be differentially affecting the expression of the investigated ferroptosis-related proteins. Finally, the DSP analysis revealed significant upregulation of 13 and downregulation of 2 ferroptosis-related genes, further supporting the hypothesis that α-syn pathology in PD is associated with a shift towards increased ferroptosis vulnerability. One of the upregulated genes in neurons from PD brains compared to controls was mitochondrial ferritin (FTMT). At the protein level, α-syn-positive neurons in PD brains exhibited lower ferritin levels compared to neurons in control brains. While this may seem contradictory, it could be explained by the higher accumulation of free iron in PD neurons, indicating that ferritin protein levels may be insufficient to store the excess iron. This imbalance might make the cells more vulnerable to ferroptosis, triggering a compensatory mechanism to upregulate ferritin gene expression. However, this upregulation does not appear to fully translate into increased protein levels, potentially due to impaired protein translation or heightened ferritin degradation in PD neurons. Furthermore, this discrepancy may also be influenced by the specificity of the LS-B14992 antibody used for ferritin detection, which is not mitochondria-specific, whereas FTMT is a mitochondrial ferritin protein. These findings suggest that analyzing cells with varying levels of α-syn aggregates could offer valuable insights into how ferroptosis-related marker expression changes at different stages of disease progression or inclusion formation. This would help elucidate the relationship between α-syn pathology, iron dysregulation, and ferroptosis vulnerability in PD.

Interestingly, it was recently reported that subregions of the SNc are not equally affected in PD and show distinct molecular and spatial transcriptomic profiles in an LRRK2 PD mouse model [53]. Similarly, our analysis of the six ferroptosis-related markers within subregions of the human SNc revealed a non-uniform vulnerability to ferroptosis across the SNc of controls. By dividing the SNc into three subregions according to their connectivity patterns and function [54], we observed that the medial SNc had an overall higher pixel positivity across all markers, except for GPX4, compared to lateral and/or ventral SNc. Higher ferritin and NCOA4 could be indicative of an elevated demand for iron storage, paralleled by increased upregulation of ferritinophagy by NCOA4 [55]. Moreover, higher 4HNE expression indicates a higher ferroptosis vulnerability in nigral neurons in the medial subregion [56]. Higher expression of ferroportin in the medial subregion could be related to a compensatory mechanism for increased intracellular iron, which is naturally elevated in the SN of healthy aged individuals [57]. It has also been reported that neurons from the dorsomedial region of healthy-aged controls had higher neuromelanin content compared to the ventrolateral SNc [58]. Neuromelanin accumulates with ageing, and it is influenced by the oxidation of dopamine during its metabolism [59]. Thus, although neuromelanin can have a long-term neuroprotective effect [60], in ageing, where it reaches its peak accumulation, neuromelanin-rich dopaminergic neurons may become more vulnerable to oxidative damage due to dopamine synthesis [59]. Therefore, increased levels of neuromelanin in medial SNc could underline its selective susceptibility to ferroptosis-related changes as a result of increased oxidative stress, compared to ventrolateral SNc. The high cytochrome c expression in medial nigral dopaminergic neurons further supports this hypothesis, as this apoptosis product is released from dysfunctional mitochondria during increased oxidative stress [14].

Importantly, to conduct a fair assessment across markers with little heterogeneity in the populations studied, within the investigation of PD neurons with age-matched controls, only ventral SNc cells were used within controls, and in PD cases, primarily ventral (although sometimes supplemented with cells from medial and lateral SNc - Supplementary Fig. 3B). This remains a limitation because in PD there is extensive neurodegeneration and neuronal loss which means that staying restricted to the ventral subarea was not always possible and cells had to be selected from one of the other two subregions. Although the lateral SNc is particularly relevant to study PD given its somatomotor function, it is also predominantly affected in disease, thereby leading to higher neuronal loss in this area [59]. Therefore, we focused on the ventral subregion as it encodes motor impulsivity [59], also relevant to PD, and other disease-related neurodegeneration.

Based on the human data, we hypothesized that the presence of α-syn fibrils in conditions of stimulated ferroptotic pathway will increase neurons’ vulnerability to cell death. This was confirmed using mouse neuronal cells and human dopaminergic cells. Interestingly, human dopaminergic cells showed higher susceptibility to RSL3-induced cell death compared with PCNs. This was in line with current literature showing that LUHMES neurons exhibited 50-fold heightened susceptibility to erastin compared to undifferentiated LUHMES cells, differentiated SH-SY5Y cells, or Neural Stem Cells (NSCs) [37]. The distinct and increased vulnerability of the dopaminergic LUHMES cells, which are derived from the mesencephalon, when compared to cortical neurons, could be attributed to their different molecular compositions, gene expressions, and signaling pathways, which could affect their response to RSL3 or other ferroptotic stimuli [36].

Beyond the substantia nigra pars compacta (SNc), emerging evidence suggests that other brain regions implicated in Parkinson’s disease (PD) may also be vulnerable to ferroptosis. Several studies have reported regional differences in ferroptosis-related markers across both PD and other neurodegenerative conditions, supporting the notion of spatial heterogeneity in ferroptotic sensitivity [61–63]. Our own in vitro data reinforce this idea by showing cell-type-specific responses to ferroptotic stress in two distinct neuronal populations. Consistent with this, degeneration of locus coeruleus noradrenergic neurons was shown to increase ferroptosis susceptibility and contribute to memory impairment in a paraquat and maneb-induced mouse model of PD [64]. Supporting regional vulnerability further, another study reported that GPX4 is present within dystrophic dopaminergic axons in the putamen of PD brains, suggesting increased ferroptotic stress in this region as well [42]. Moreover, while iron accumulation in the SN is a well-known hallmark of PD pathology, it has also been observed in the caudate nucleus and pallidum during normal aging [65]. Although our study primarily focuses on the TH-positive SNc neurons, these findings collectively highlight the need to explore region- and cell-specific vulnerability to ferroptosis in order to better understand the disease mechanisms and optimize future therapeutic strategies.

In this study, by targeting the ferroptotic pathway using two different inhibitors, Fer-1 but not DFO, we showed that they can mediate neuroprotection in conditions of cell death induced by α-syn and an initiator of ferroptosis, RSL3. We showed that pre-treatment with Fer-1 but not DFO could protect against α-syn-induced cell death in cortical neurons. However, only Fer-1 induced neuroprotection in human differentiated dopaminergic cells. Fer-1 directly targets and inhibits lipid peroxidation, while DFO acts by chelating iron, which indirectly prevents lipid peroxidation and subsequent ferroptosis. Clinically, it has been described that the administration of DFO has been linked to various systemic toxicities, including adverse effects on the cardiovascular, respiratory, gastrointestinal, cutaneous, and nervous systems [66]. In a previous study, we tested the neuroprotective efficacy of another iron chelator, ethyl-3,4-dihydroxybenzoate (DHB), in an overexpression of wt α-syn model of PD in human dopaminergic neurons, and observed that, similarly to DFO, DHB did not prevent α-syn-mediated cell death [67]. Interestingly, DFO and DHB could prevent glutamate-induced ferroptotic cell death in neuronal HT22 cells [17], but their neuroprotective effects were not observed in human dopaminergic cells. These findings suggest that targeting lipid peroxidation might provide more rescue effects on α-syn-related pathology than iron chelation in human dopaminergic neurons. However, targeting both iron homeostasis and lipid peroxidation aspects of the ferroptosis mechanism at the same time might provide beneficial effects in mitigating α-syn pathology.

Using the α-syn overexpression model in *C. elegans* provided an excellent opportunity to test our hypothesis in vivo [30]. We found that α-syn overexpression led to doubling in iron levels in *C. elegans* compared to worms without α-syn, and that the number of α-syn inclusions was reduced by both iron chelation and lipid peroxidation inhibition. Recent studies suggest that ferroptosis is the primary driver of iron-overload-mediated damage and that oleic acid can inhibit iron-overload-induced ferroptotic damage in *C. elegans* [68]. Another study supports this by showing that aging and glutathione depletion-related iron overload lead to ferroptotic cell death in *C. elegans* [69]. Additionally, *ftn-1* encoded ferritin was found to be elevated and identified as an essential mediator of longevity, suggesting that protecting cells by storing iron and reducing free available iron, which could induce higher vulnerability to ferroptosis, has a beneficial effect on aging [70]. Our data further support the link between ferroptosis and aging, and add more specific evidence that age-related neurodegenerative diseases [15, 71], such as Parkinson’s, may be mitigated by ferroptosis inhibition. However, the effects on α-syn were not linked to changes in motility. This could be explained by a recent study showing that α-syn might not necessarily be associated with toxicity, as a proxy for the evaluation of the motility in *C. elegans* [39]. In addition, the observed reduction in α-syn inclusions by ferroptotic inhibitors is significant but still minor, which might not be enough to translate into differences in worm motility.

In conclusion, these results contribute to the growing body of knowledge on the involvement of the ferroptosis pathway in PD pathology, particularly α-syn-related pathology. The findings show that α-syn fibrils increase vulnerability to ferroptosis, highlighting the potential for targeting this cell death pathway as a therapeutic strategy for PD while recognizing that other forms of cell death, such as apoptosis and necrosis, also co-exist. Further research investigating the causal relationship between α-syn and the ferroptosis mechanism in the context of PD is necessary to bridge the gap in developing neuroprotective treatments against lipid peroxidation that could delay or halt the progression of PD pathology.

## Supplementary information

Supplementary tables (all)

Supplemental material

## Data Availability

The DSP experiment forms part of a broader ongoing project within our laboratory. For the current study, we present results from the analysis focusing on ferroptosis-related genes. The corresponding gene list and associated *p*-values (significant or not) are provided in Supplementary Table 6. The underlying DSP dataset and analytical code are part of the same ongoing project and will be made available upon completion of the related studies.
