# Supplementary material for: Ferroptosis inhibition protects against α-synuclein-related neuronal cell death
Source: Cell Death Dis. 2025 Dec 14;17(1):78. doi: 10.1038/s41419-025-08319-z (PMC12827279; doi:10.1038/s41419-025-08319-z)
Supplement: Supplementary file 2 — Supplemental material [file 41419_2025_8319_MOESM2_ESM.docx]

**Supplementary material**


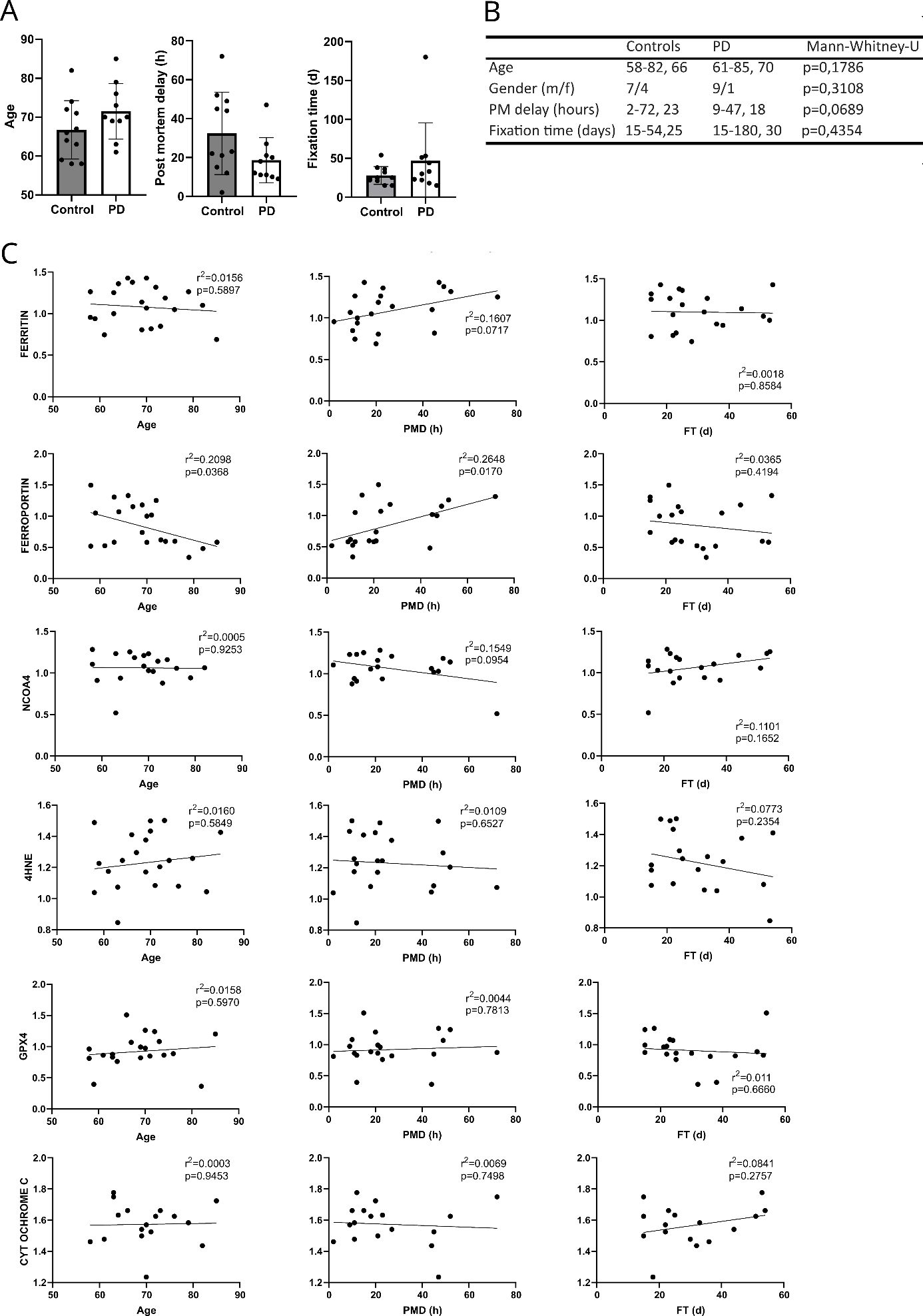


***Supplementary figure 1:*** *Covariate effects on protein expression of ferroptosis-related markers in single cells located in SN of control and PD patient’s brain.* ***A.*** *Differences between controls and subjects with PD in age (m=male, f=female), post mortem delay (PMD) (in hours) and fixation time (FT) (in days). B. Table summarizing the range of age, gender PMD and FT between controls and subjects with PD. Numbers in second and third column are reported as: min-max, median. No significant differences were observed between controls and PD subjects (Mann-Whitney U test, p<0.05).* ***C.*** *A scatter plot with simple linear regression illustrating the level of correlation between age, PMD and FT and each ferroptosis-related marker expression, along with the line of best fit. No real correlation between the ferroptosis-related markers' expressions with any of the studied variables has been observed (Spearmans's correlation test and simple linear regression, r^2^=0.0003-0.2648, p=0.0170-p=0.9453).*


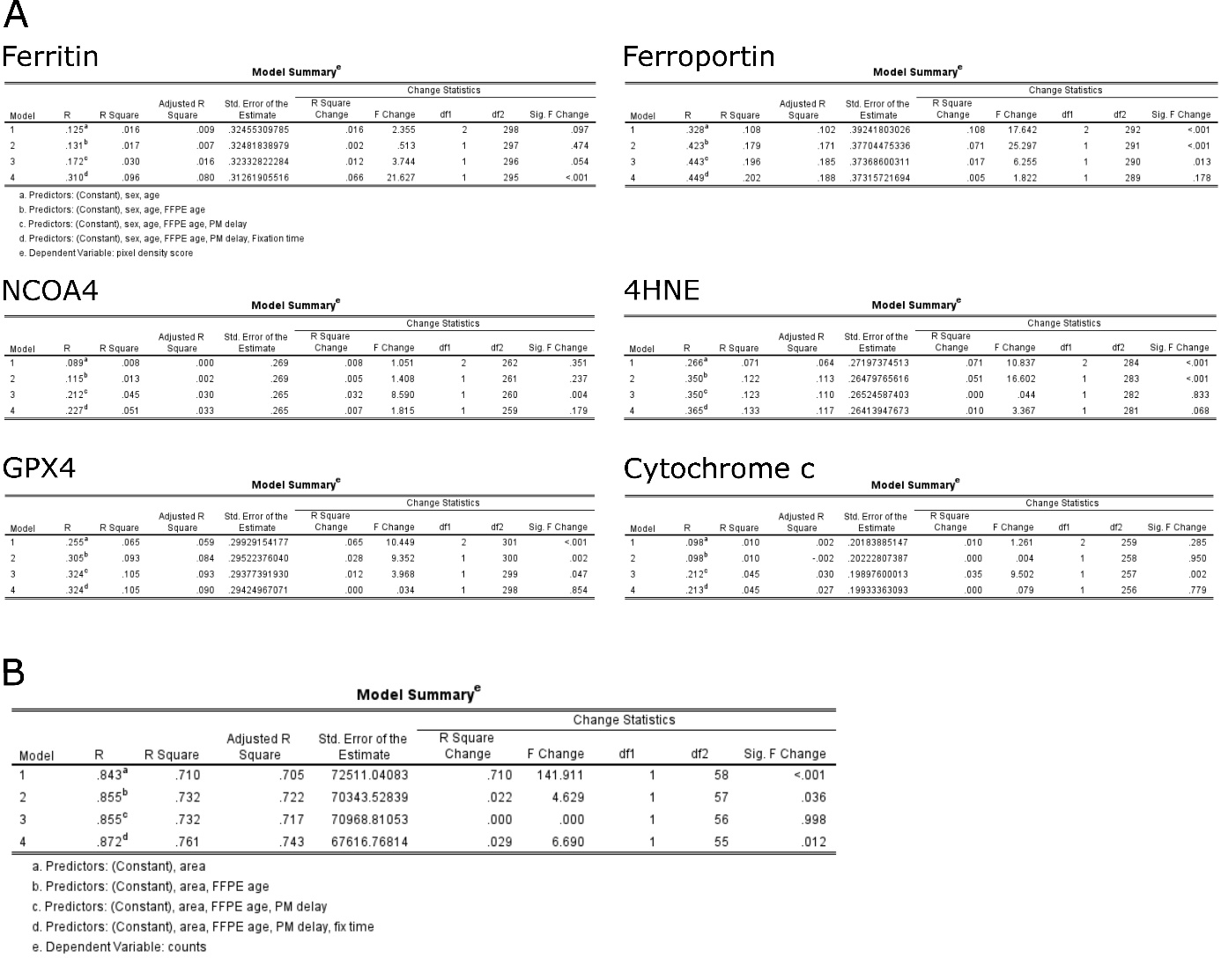


***Supplementary figure 2****: Multiple linear regression (MLR) results to evaluate the effects of tissue variables on ferroptosis-related outcomes.* ***A****. Tables summarizing the output of the MLR showing the explained variability of each ferroptosis-related marker expression based on hierarchical predictors: age and sex of the patient, age of the formalin-fixed paraffin-embedded (FFPE) block, post-mortem (PM) delay and fixation time. The addition of these variables to the model only significantly explained a maximum of 18.5% variability in pixel density scoring values.  Maximum variability changes are reported at 6.6% in ferritin (p<0.001), 10.8% in ferroportin (p<0.001), 3.2% in NCOA4 (p=0.004), 7.1% in 4HNE (p<0.001), 6.5% in GPX4 (p<0.001), 3.5% in cytochrome c (p=0.002).* ***B****. Table summarizing the output of the MLR showing the explained variability of mRNA counts based on hierarchical predictors: area positive for tyrosine hydroxylase (TH), age of FFPE block, PM delay and fixation time.  TH-positive area significantly explains 70.5% (p<0.001) of the variability observed in the unique mRNA counts obtained from PD and control patients. Age of the formalin-fixed, paraffin embedded (FFPE) block, and fixation time only increased the (adjusted) R-squared values to 0.722 (p=0.036) and 0.743 (p=0.012), respectively. Post-mortem delay does not significantly increase the predictability of the number of mRNA counts (p=0.998). Unique mRNA counts in the cohort are impacted by the TH-positive area selected for sequencing, and likely not affected by variables pertaining to the tissue quality.*


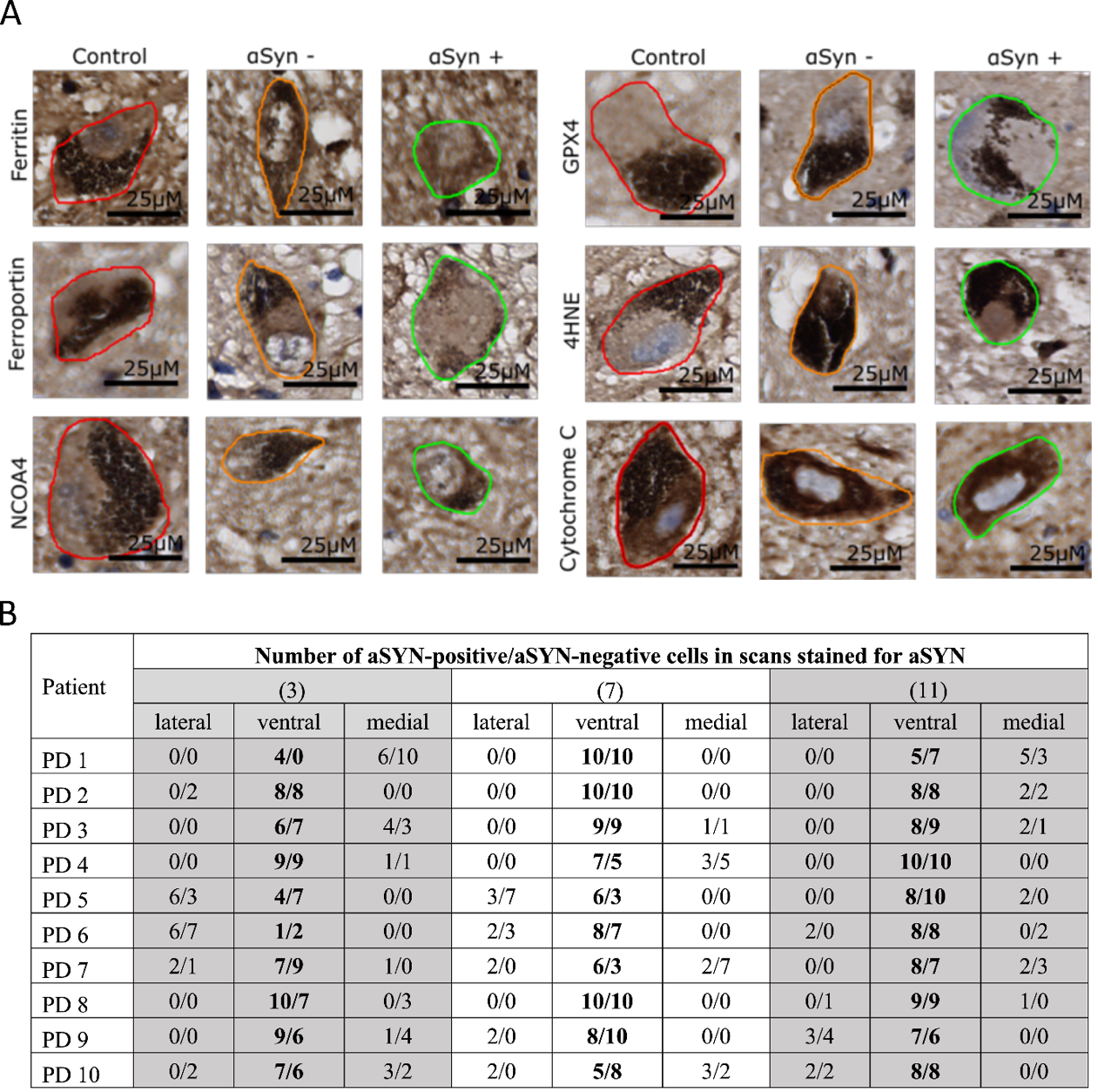


***Supplementary figure 3:*** Representative images of neurons used in immunohistochemistry, and SN subregion cell selection in PD cases. **A**. *Example of the expression of ferroptosis related markers in single cells from control and PD brain. A visual representation of how we defined individual cells, facilitating positive pixel density analysis of protein expression stains. We examined the protein expression of crucial ferroptosis-related markers, including ferritin, ferroportin, NCOA4, GPX4, 4HNE, and cytochrome c, within single cells. These cells were obtained from three distinct groups: Control (cells from the brain of aged-matched individuals without Parkinson's Disease (PD), serving as a baseline reference group), α-syn positive cells (cells showing the presence of α-syn in PD patients), and α-syn negative cells from PD Patients (cells from PD patients lacking α-syn expression).* ***B****. Table showing the ratio of alpha-synuclein (aSYN)-positive/-negative neurons selected from each subregion of the substantia nigra pars compacta, across all three aSYN stained slides in the 10 patients included. The selection involved (at least) 10 aSYN-positive and 10 aSYN-negative cells per patient, per aSYN-stained slide; this was not possible for one of the scans of patient PD 6 due to extensive neurodegeneration. Most cells were selected from the ventral area, but oftentimes supplemented with some cells from lateral and/or given the extensive neurodegeneration and consequent inability to find all necessary cells restricted to one subregion.*

***
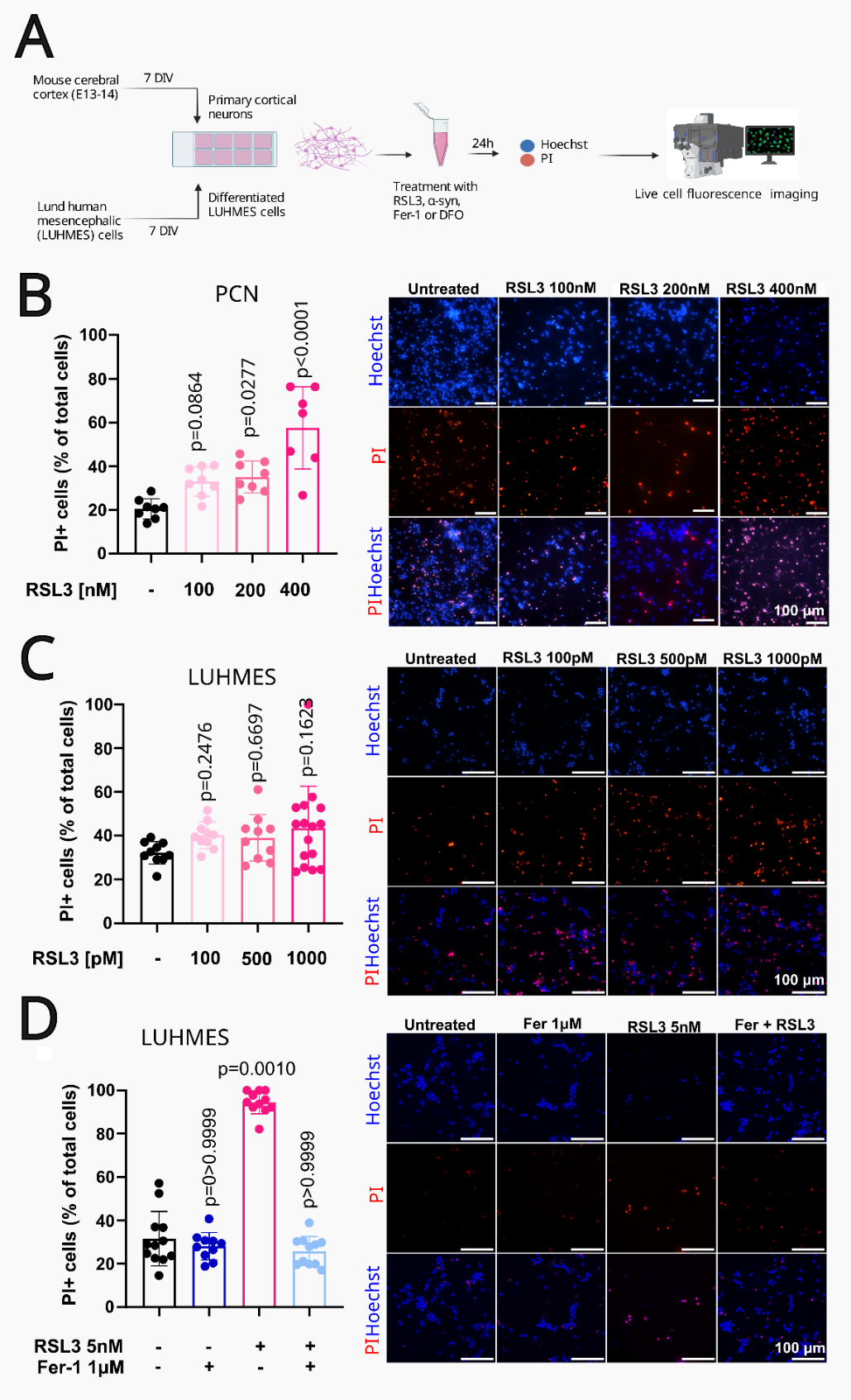
***

***Supplementary figure 4: RSL3 challenge in dopaminergic neurons and cortical neurons. A.*** *Overview of the experimental design.* ***B.*** *PI uptake in PCNs treated with RSL3 (100, 200 and 400 nM). Quantification of PI-positive cells in relation to total cells of PI uptake in response to various RSL3 concentrations (left) and representative images (right).* ***C.*** *PI uptake is increased in LUHMES cells treated with RSL3 (100, 500, and 1000pM) in a concentration-dependent manner. LUHMES cells were treated with RSL3 for 24hrs. Quantification of PI-positive cells in relation to total cells treated with RSL3 (left) and representative images (right).* ***D.*** *5nM RSL3 caused high % of PI-positive cells. Not plotted 10 and 50nM RSL3 as the PI uptake visualized while imaging was close to 100%. This increase was prevented by 1µM Fer-1 pre-treatment for 4h. Fer-1 was present as well during the challenge with RSL3 for another 24h. Each graph in this figure is a representative experiment (7-12 technical replicates) from a total of three biological replicates. Each replicate was conducted independently to ensure the robustness and consistency of our results and similar trends were observed in the remaining two replicates. Statistical significance was determined by Kruskal Wallis with Dunn’s post-hoc multiple comparison (p<0.05).*

*
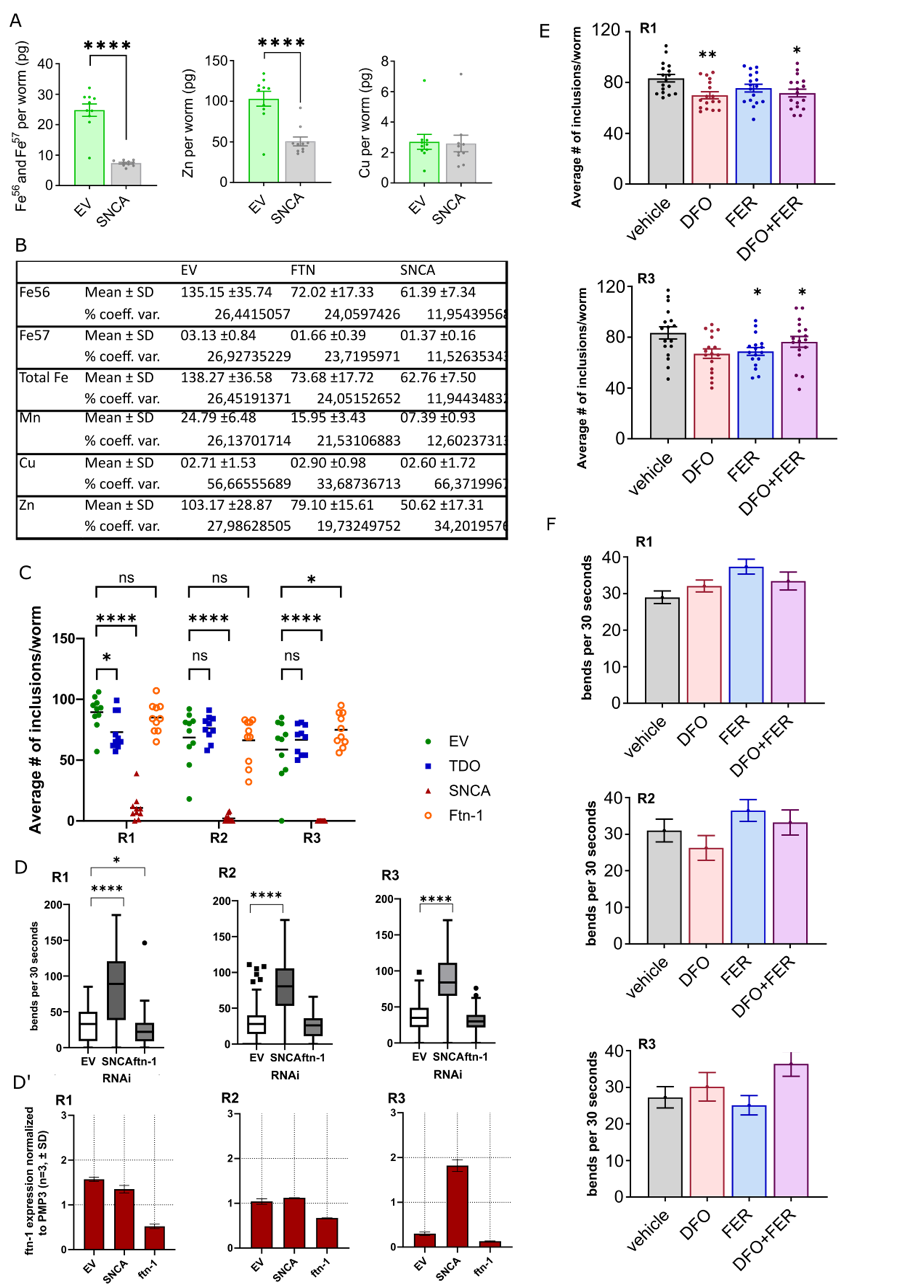
*

***Supplementary figure 5:*** *Supporting data for experiments on the C. elegans α-syn overexpression model.* ***A.*** *α-syn presence can be linked to significantly higher levels of various metals including manganese (Mn) and zinc (Zn) in C. elegans α-syn overexpression model (empty vector, EV) compared to when RNAi of α-synuclein (SNCA) is present. There was no difference in copper levels in between these two models. Each graph represents a representative example out of 3 independent experiments. n=10, 200 worms/genotype, ^****^p<0.0001.* ***B.*** *Elemental content presented as mean pg per worm ± standard deviation with n=10 per genotype. Percent coefficient of variation (CoV) shown for each genotype. Elements: Mn, Zn, Cu and iron (Fe56, Fe57, and total iron). Groups: EV, SNCA and in ftn-1 knockdown (FTN).* ***C.*** *The difference in average number of α-syn inclusions per worm in 3 independent experiments between groups: EV, 2,3‐dioxygenase (TDO), SNCA and in Ftn-1 knockdown. As expected, upon knockdown of the α-syn expression, almost no remaining inclusions were detected and the worms showed an improved locomotion (Supplementary figure 3C, D). Knocking down ftn-1 led to an increase in the number of α-syn inclusions in one experiment (Supplementary figure 3C, replicate 3) and a decrease in motility in another experiment (Supplementary figure 3C, replicate 1) but these effects were only observed in one out of three independent experiments and were not consistently reproducible.* ***D.*** *Analysis of motility in EV, SNCA and in Ftn-1 knockdown α-syn overexpressing worms showing a consistent trend of decreased motility in but these changes were significant only in 1/3 experiments.* ***D’.*** *qPCR results of ftn-1 expression per corresponding replicate.*  ***E.*** *Analysis of motility in α-syn overexpressing worms (OW40) treated with vehicle (DMSO2%), deferoxamine (DFO), ferrostatin-1 (Fer-1) and a combination of DFO and Fer-1 at the larval stage 4 (L4). No changes were observed between groups.* ***F.*** *The other two repeats of inclusion analysis in α-syn overexpressing worms treated with vehicle (DMSO2%), DFO, Fer-1 and a combination of DFO and Fer-1. Statistical significance was determined by a t-test or one-way ANOVA with multiple comparisons (p<0.05).*
